# Defining the root cause of reduced H1N1 live attenuated influenza vaccine effectiveness: low viral fitness leads to inter-strain competition

**DOI:** 10.1038/s41541-021-00300-z

**Published:** 2021-03-12

**Authors:** Oliver Dibben, Jonathan Crowe, Shaun Cooper, Laura Hill, Katarzyna E. Schewe, Helen Bright

**Affiliations:** Flu-BPD, Biopharmaceutical Development, R&D, AstraZeneca, Liverpool, UK

**Keywords:** Live attenuated vaccines, Influenza virus

## Abstract

In the 2013–14 and 2015–16 influenza seasons, reduced vaccine effectiveness (VE) was observed for the H1N1 component of the FluMist quadrivalent live attenuated influenza vaccine (QLAIV) in the USA, leading to loss of Advisory Committee on Immunization Practices recommendation. Here we demonstrate in ferrets that 2015–16A/H1N1pdm09 vaccine strain A/Bolivia/559/2013 (A/BOL13) is outcompeted in trivalent (TLAIV) and QLAIV formulations, leading to reduced protection from wild-type challenge. While monovalent (MLAIV) A/BOL13 provided significant protection from wild-type virus shedding and fever at doses as low as 3.0 log_10_ fluorescent focus units (FFU), it failed to provide a similar level of protection in TLAIV or QLAIV formulation, even at a 6.0 log_10_ FFU dose. Conversely, clinically effective H1N1 strain A/New Caledonia/20/1999 provided significant protection in MLAIV, TLAIV, and QLAIV formulations. In conclusion, reduced A/BOL13 replicative fitness rendered it susceptible to inter-strain competition in QLAIV, contributing to its reduced VE in the 2015–16 season.

## Introduction

FluMist/Fluenz^®^ is an intranasal live attenuated influenza vaccine (LAIV), with vaccine virus replication occurring in the epithelia of the upper respiratory tract, resulting in generation of both humoral and cell-mediated immune responses^1–3^.

In the 2013–14 influenza vaccine season, the influenza A pandemic 2009 H1N1 (A/H1N1pdm09) component of quadrivalent LAIV (QLAIV) was found to have low vaccine effectiveness (VE) in the USA^4,5^. The initial hypothesized root cause for this low VE was low thermostability of the hemagglutinin (HA) protein of the A/H1N1pdm09 strain, A/California/07/2009 (A/CA09)^6,7^. A/CA09 was subsequently replaced with a more thermostable strain, A/Bolivia/559/2013 (A/BOL13). However, A/BOL13 demonstrated mainly low–moderate effectiveness in the 2015–16 season^5^, with high VE (65%) only seen in a single study^8^. Recent studies showed that A/BOL13 possessed HA thermal and pH stability profiles comparable with historic, clinically effective LAIV strains^9^, suggesting that factors other than HA stability were contributing to the reduced A/H1N1pdm09 VE.

As a result of this reduced VE against H1N1 in the 2013–14 and 2015–16 seasons, the US Advisory Committee on Immunization Practices (ACIP) recommended that QLAIV not be used for the 2016–17 and 2017–18 seasons^10^. A series of investigations was subsequently initiated to identify the root cause of this reduced VE and facilitate identification of more effective strains.

Initially, retrospective analyses of clinical data suggested that the VE of an individual A/H1N1pdm09 LAIV strain might be affected by varying vaccine strain composition. The 2013–14 H1N1 strain A/CA09 generated variable VE in different seasons^5,8^, being clinically effective in monovalent LAIV (MLAIV) form during the 2009 pandemic and in a trivalent LAIV (TLAIV) formulation in Canada in 2013–14, but showing low VE in TLAIV formulation in the USA in 2010–11, similar to the 2013–14 USA QLAIV. By contrast, during randomized controlled clinical trials, a pre-2009 seasonal H1N1 LAIV strain, A/New Caledonia/20/1999 (A/NC99), provided high efficacy levels in multiple TLAIV vaccine formulations^11–15^.

In vitro investigations then found that post-pandemic A/H1N1pdm09 replicative fitness in fully differentiated primary human nasal epithelial cells (hNEC) was significantly reduced relative to pre-2009 LAIV strains^16^. This provided a plausible root cause for a generalized reduction in A/H1N1pdm09 VE. It also raised the possibility that less fit A/H1N1pdm09 strains could be outcompeted in multivalent formulations, providing a putative mechanism for composition-dependent reductions in VE. However, there was no in vivo evidence that reduced replicative fitness was responsible for reduced effectiveness and that the effect could be influenced by inter-strain competition.

Consequently, we investigated whether the less fit 2015–16A/H1N1pdm09 strain A/BOL13 would provide inferior protection relative to fitter pre-2009 strain A/NC99. In addition, we assessed whether the reduced fitness of A/BOL13 would render it more susceptible to inter-strain competition in TLAIV and QLAIV formulations than A/NC99, providing a mechanism for its reduced clinical VE in QLAIV. Here, we present the findings from this crucial work identifying a direct role for reduced replicative fitness in low and variable A/H1N1pdm09 VE, informing subsequent selection of H1N1 LAIV strains for commercial vaccine formulations.

## Results

### A/BOL13 LAIV shedding is reduced relative to A/NC99

**Fig. 1:**
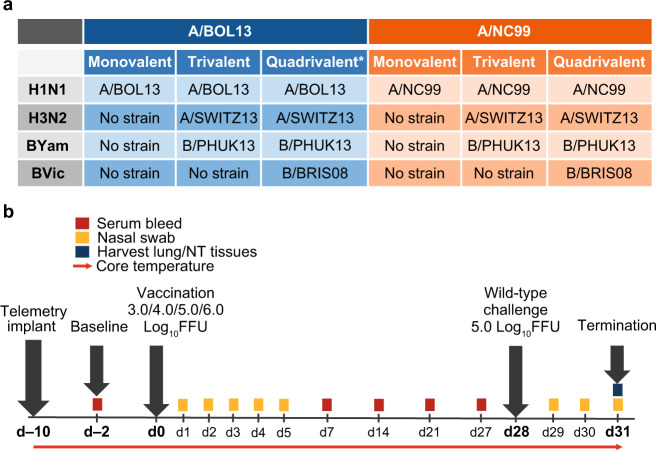
Study schedule for comparison of H1N1 LAIV of varying fitness in ferret model. **a** Composition of vaccine formulations. Rows detail subtype composition. n/a indicates no strain was present for that subtype. **b** Ferrets were intranasally vaccinated at Study Day 0 with monovalent, trivalent, or quadrivalent LAIV formulations over a range of doses. Gray arrows indicate major interventions over the 31-day study course. Colored boxes highlight sampling points, with time in days on horizontal axis. Red arrow indicates hourly telemetry recording of core body temperature. *Representative of 2015–16 commercial formulation. d day, A/BOL13 A/Bolivia/559/2013, A/NC99 A/New Caledonia/20/1999, A/SWITZ13 A/Switzerland/9715293/2013, B/BRIS08 B/Brisbane/60/2008, B/PHUK13 B/Phuket/3073/2013, FFU fluorescent focus units, LAIV live attenuated influenza vaccine, NT nasal turbinate.

To assess the importance of H1N1 fitness differences in terms of in vivo efficacy, ferrets were vaccinated with either A/BOL13 or A/NC99 in MLAIV, TLAIV, and QLAIV formulations. Initially, groups of four animals were vaccinated with 3.0, 4.0, 5.0, or 6.0 log_10_ fluorescent focus units (FFU)/strain and nasal swabs were taken daily for 5 days (Days 1–5) post-vaccination to assess vaccine virus shedding (Fig. 1). To reduce ferret numbers, a human 7.0 log_10_ FFU dose group was not included, instead focusing on lower doses with the aim of identifying limits of protection.

**Fig. 2:**
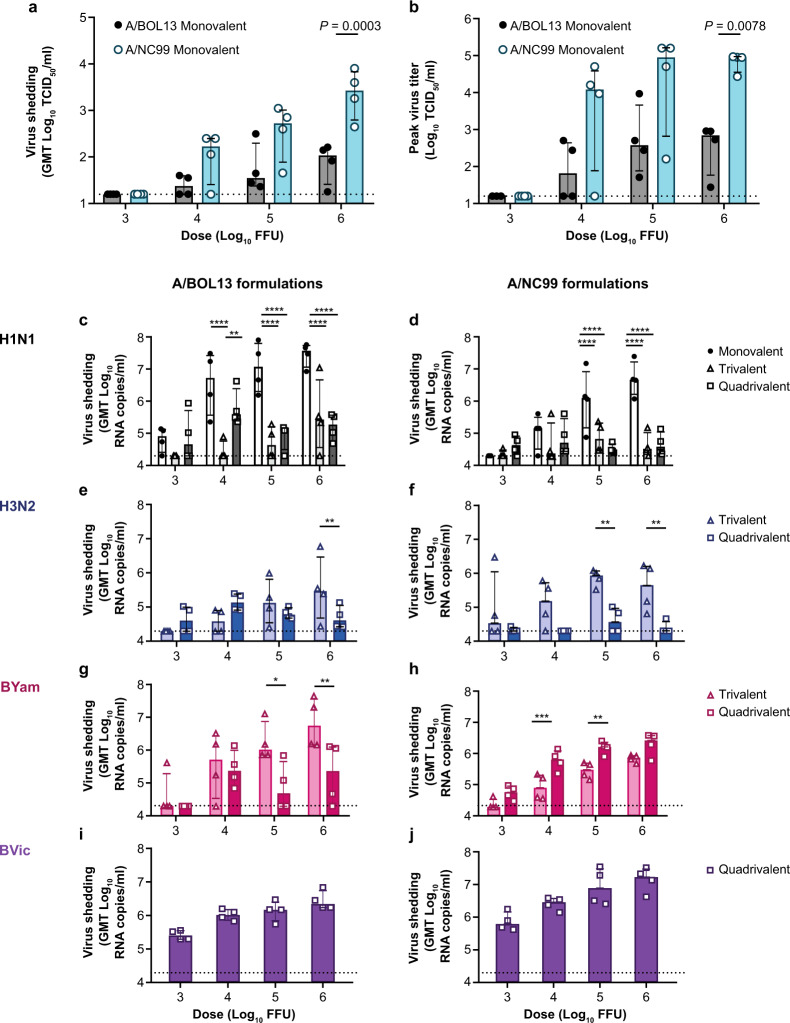
Monovalent A/BOL13 shedding is reduced relative to A/NC99 and all H1N1 virus shedding is inhibited in multivalent formulations. Comparison of virus shedding for all strains in A/BOL13 and A/NC99 formulations. **a** Geometric mean Day 1–5 shedding of monovalent LAIV H1N1 strains, measured by TCID_50_. **b** Monovalent LAIV H1N1 peak titer post-vaccination. **c**–**j** Shedding of all strains in monovalent, trivalent, and quadrivalent LAIV formulations, measured by multiplex RT-qPCR. Virus shedding in A/BOL13 formulations are shown in **c** (A/BOL13), **e** (A/SWITZ13), **g** (B/PHUK13), and **i** (B/BRIS08). Virus shedding in A/NC99 formulations are shown in **d** (A/NC99), **f** (A/SWITZ13), **h** (B/PHUK13), and **j** (B/BRIS08). Geometric mean virus titers for individual animals are indicated by the circle, triangle, and square symbols on the graphs, with 4 animals per dose group. Columns and error bars for all virus titer data show group median and interquartile range. Statistical comparison between groups was performed by two-way analysis of variance with Sidak’s post-test correcting for multiple comparisons. *P < 0.05, **P < 0.01, ***P < 0.001, ****P < 0.0001. Dotted lines indicate lower limit of detection; values below this were plotted as 0.5× the lower limit of detection. A/BOL13 A/Bolivia/559/2013, A/NC99 A/New Caledonia/20/1999, A/SWITZ13 A/Switzerland/9715293/2013, B/BRIS08 B/Brisbane/60/2008, B/PHUK13 B/Phuket/3073/2013, GMT geometric mean titer, LAIV live attenuated influenza vaccine, RT-qPCR quantitative reverse transcription-polymerase chain reaction, TCID_50_ tissue culture infectious dose 50%.

Infectious virus shedding by A/NC99 and A/BOL13 were expressed as the geometric mean of Days 1–5 tissue culture infectious dose 50% (TCID_50_) titers. This utilized all available timecourse data to produce a single data point per animal, for statistical comparison. These data clearly showed that MLAIV A/NC99 produced more infectious virus than MLAIV A/BOL13 (Fig. 2a). However, this was only statistically significant in the 6.0 log_10_ FFU dose group (*P* = 0.0003). Comparison of peak virus titers corroborated this (Fig. 2b), with A/NC99 reaching peak shedding levels 100-fold higher than those of A/BOL13 (*P* = 0.0078). This confirmed the A/BOL13 replicative fitness deficiency previously observed in hNEC^16^. Interestingly, while MLAIV A/NC99 shed significantly more infectious virus than MLAIV A/BOL13, viral RNA (vRNA) shedding for A/BOL13 was higher than that of A/NC99 (Supplementary Fig. 1a). Calculating the ratio of total virus (vRNA copy number) to infectious virus (TCID_50_ titer) in individual MLAIV nasal swabs showed that A/BOL13 generated 100-fold more non-infectious virus than A/NC99 (*P* < 0.0001, Supplementary Fig. 1b), providing a potential explanation for its lower fitness.

All LAIV shedding kinetics are shown in Supplementary Figs. 2–4. These data indicated no or delayed shedding of fully infectious virus for both MLAIV A/BOL13 and A/NC99 at 3.0 and 4.0 log_10_ FFU doses, relative to 5.0 or 6.0 log_10_ FFU. In addition, the trend for MLAIV A/BOL13 vRNA shedding in the absence of infectious virus could be seen across the timecourse at both 3.0 and 4.0 log_10_ FFU doses. A/NC99 showed a clearer agreement between TCID_50_ and quantitative reverse transcription-polymerase chain reaction (RT-qPCR) kinetics at all MLAIV doses.

In TLAIV and QLAIV formulations, shedding of vRNA for both A/BOL13 and A/NC99 was reduced relative to MLAIV (an exception was the 3.0 log_10_ FFU dose for A/NC99), with statistically significant reductions of ~100-fold seen at 5.0 and 6.0 log_10_ FFU doses (Fig. 2c and d). However, as RT-qPCR titers for both strains approached the limit of detection, comparison of the reduction in replication of each strain in TLAIV and QLAIV relative to MLAIV was not possible.

Shedding of H3N2 and B subtypes in TLAIV and QLAIV formulations was also measured. H3N2 A/Switzerland/9715293/2013 (A/SWITZ13) geometric mean Days 1–5 vRNA shedding was detectable in A/BOL13 formulations, with no statistically significant differences between TLAIV or QLAIV except for the 6.0 log_10_ FFU dose (Fig. 2e). However, in A/NC99 formulations, A/SWITZ13 shedding was undetectable in most QLAIV-vaccinated animals (Fig. 2f). B/Phuket/3073/2013 (B/PHUK13) and B/Brisbane/60/2008 (B/BRIS08) shed more robustly than either A subtype in TLAIV and QLAIV (Fig. 2g–j), with B/BRIS08 producing the highest levels of vRNA in QLAIV-vaccinated animals (Fig. 2i and j).

### A/BOL13 serum immune responses are reduced relative to A/NC99

Serum antibody responses were characterized by microneutralization (MN) assays at Days 7, 14, 21, and 27 post-vaccination. By Day 14, antibody responses had peaked for all subtypes and were sustained through Day 27 (Supplementary Fig. 5). A single representative timepoint, Day 21, was then selected to describe responses in individual ferrets by both MN and HA inhibition (HAI) assays.

**Fig. 3:**
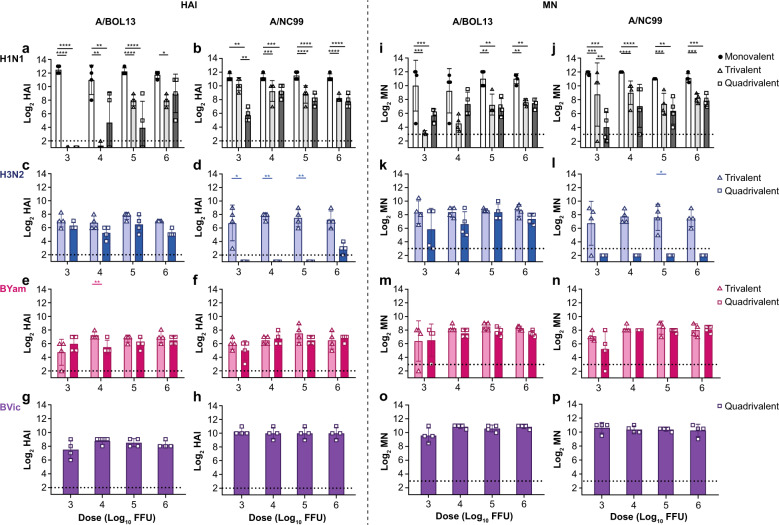
A/BOL13 serum immune responses are more strongly reduced in multivalent formulations. Serum immune responses were measured by HAI and MN assays at Days 7, 14, 21, and 27 post-vaccination. Representative data from Day 21 are shown. **a**, **b** HAI and MN data for all strains in A/BOL13 containing formulations. HAI—**a** (A/BOL13); **c** (A/SWITZ13); **e** (B/PHUK13); **g** (B/BRIS08). MN—**b** (A/BOL13); **d** (A/SWITZ13); **f** (B/PHUK13); **h** (B/BRIS08). **i**–**p** HAI and MN data for all strains in A/NC99 containing formulations. HAI—**i** (A/NC99); **k** (A/SWITZ13); **m** (B/PHUK13); **o** (B/BRIS08). MN—**j** (A/NC99); **l** (A/SWITZ13); **n** (B/PHUK13); **p** (B/BRIS08). Bar graphs show geometric mean log_2_ titers for individual animals (symbols), 4 animals per dose group. Columns and error bars show group geometric mean and group geometric standard deviation. Statistical comparison in all cases was performed by two-way analysis of variance with Sidak’s post-test correcting for multiple comparisons.*P < 0.05, **P < 0.01, ***P < 0.001, ****P < 0.0001. Dotted lines indicate lower limit of detection; values below this were plotted as 0.5× the lower limit of detection. A/BOL13 A/Bolivia/559/2013, A/NC99 A/New Caledonia/20/1999, A/SWITZ13 A/Switzerland/9715293/2013, B/PHUK13 B/Phuket/3073/2013, B/BRIS08 B/Brisbane/60/2008, FFU fluorescent focus units, HAI hemagglutination inhibition, MN microneutralization.

Both MLAIV A/BOL13 and MLAIV A/NC99 produced similarly high (10–12 log_2_) serum antibody titers by both HAI and MN (Fig. 3). However, while all A/NC99 MLAIV-vaccinated animals produced HAI and MN responses at all vaccine doses, one of four MLAIV A/BOL13 animals in each of the 3.0 and 4.0 log_10_ FFU dose groups failed to generate neutralizing antibody responses (Fig. 3b). This indicated that, irrespective of fitness, H1N1 strains delivered in MLAIV formulation could generate consistently high levels of serum antibodies at doses as low as 5.0 log_10_ FFU. At lower doses, magnitudes of responses remained the same but occurred in fewer animals, indicating a potential discrimination between more and less fit H1N1 strains.

Compared with MLAIV, in TLAIV- and QLAIV-vaccinated animals A/BOL13 generated considerably reduced serum immune responses (Fig. 3). This was dose-dependent, with the lowest vaccine doses producing the lowest response. In 3.0 and 4.0 log_10_ FFU A/BOL13 dose groups, no TLAIV- and only two QLAIV-vaccinated animals generated detectable HAI titers, versus geometric mean HAI responses of ~10 log_2_ in MLAIV animals at the same doses. The same trend was apparent by MN, but with low levels of response detectable in low-dose groups. By contrast, while magnitudes tended to be reduced relative to MLAIV, all A/NC99 TLAIV- and QLAIV-vaccinated animals produced detectable HAI responses at all doses. Indeed, A/NC99 TLAIV HAI and MN responses were statistically indistinguishable from MLAIV in 3.0 log_10_ FFU-vaccinated animals. This indicated that A/BOL13 was able to produce comparable serum antibody responses to A/NC99 in MLAIV-responding animals but suffered a distinct reduction in serum immune responses in multivalent formulations, most notably at the lowest 3.0 and 4.0 log_10_ FFU vaccine doses. A/NC99 was considerably less affected.

In A/BOL13 formulations, H3N2 A/SWITZ13 serum antibody responses were consistent across the dose range by both HAI and MN (Fig. 3c and d). However, in A/NC99 formulations, A/SWITZ13 only generated detectable responses in TLAIV (Fig. 3k and l). B/PHUK13 and B/BRIS08 generated consistent HAI and MN responses in all vaccinated groups irrespective of formulation.

### A/BOL13 demonstrates reduced protection from influenza-like illness relative to A/NC99

**Fig. 4:**
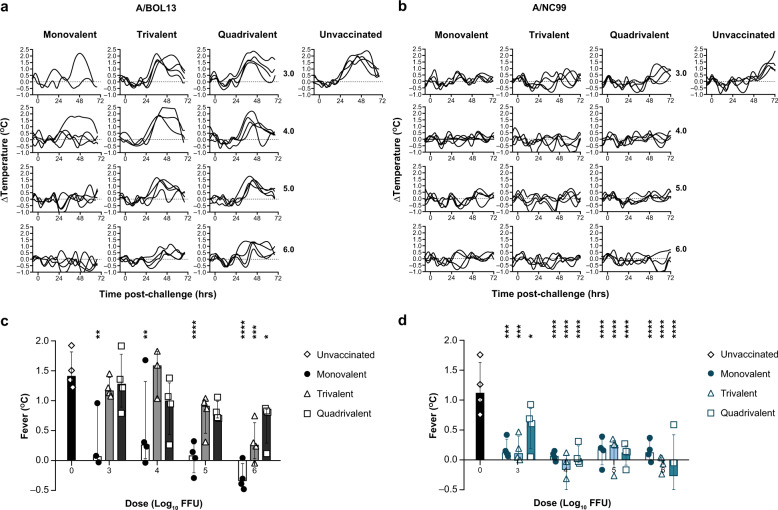
A/BOL13 provides reduced protection against influenza-like illness. Change in ferret core body temperature was measured hourly from pre-vaccination to study termination. Change in individual animal body temperature versus baseline (Δtemp [°C]) following wild-type challenge for all animals is shown in **a** (A/BOL13) and **b** (A/NC99). Curves are spline fits to hourly data. Individual fever values are summarized in **c** (A/BOL13) and **d** (A/NC99). Temperature traces from two animals in the A/BOL13, 3.0 log_10_ FFU, monovalent LAIV-dose group were corrupted and had to be excluded from this analysis. Bar graphs show fever values from individual animals (symbols), four animals per dose group. Fever temperatures shown are the average increase over baseline body temperature post-challenge, as described in “Methods”. Columns and error bars show group median and interquartile range. Statistical assessment of fever was conducted using two-way analysis of variance with Sidak’s post-test correcting for multiple comparisons. All vaccinated groups were compared with unvaccinated controls. *P < 0.05, **P < 0.01, ***P < 0.001, ****P < 0.0001. LAIV live attenuated influenza vaccine, A/BOL13 A/Bolivia/559/2013, A/NC99 A/New Caledonia/20/1999, FFU fluorescent focus units.

At Day 28 post-vaccination, all study animals were intranasally challenged with 5.0 log_10_ FFU of homologous wild-type (wt) challenge virus and development of fever was quantified as a measure of influenza-like illness (ILI). A control, unvaccinated group was included for each wt virus. Post-challenge Δtemp spline curves for A/BOL13 and A/NC99 are shown in Fig. 4a and b, along with individual fever values for statistical comparison (Fig. 4c and d). Fever temperatures shown are the average increase over baseline body temperature post-challenge, as described in the “Methods”. Temperature traces from two animals in the A/BOL13, 3.0 log_10_ FFU, MLAIV-dose group were corrupted and had to be excluded from this analysis.

Unvaccinated animals challenged with both wt A/BOL13 and wt A/NC99 developed significantly elevated body temperatures following challenge, with wt A/BOL13 temperature elevation beginning ~24 h earlier than that for wt A/NC99 (Fig. 4a and b). Median fever for unvaccinated animals was 1.43 °C for wt A/BOL13 and 1.15 °C for wt A/NC99 (Fig. 4c and d). MLAIV A/BOL13 vaccination significantly reduced fever in all treatment groups to unvaccinated levels. However, as seen for pre-challenge endpoints, a single animal in both 3.0 and 4.0 log_10_ FFU dose groups did not respond to vaccination, developing unvaccinated levels of fever. TLAIV and QLAIV A/BOL13 did not significantly reduce fever at any dose lower than 6.0 log_10_ FFU, with no indication of protection at 3.0 or 4.0 log_10_ FFU. By contrast, A/NC99 provided significant reduction in fever in MLAIV, TLAIV, and QLAIV formulations at all doses, relative to unvaccinated controls. However, the 3.0 log_10_ FFU QLAIV group did appear to provide lesser protection against fever (Fig. 4d).

These data suggested that the reduced replication and immunogenicity of A/BOL13 seen in TLAIV and QLAIV formulations led to reduced protection from ILI following wt challenge at low vaccine doses.

### A/BOL13 demonstrates reduced protection from wt virus shedding relative to A/NC99

**Fig. 5:**
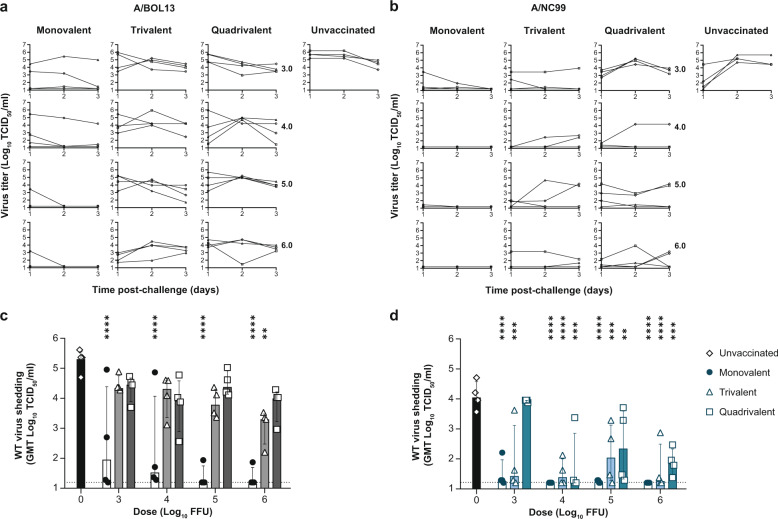
A/BOL13 provides reduced protection against wt challenge virus shedding. Shedding of homologous wt challenge virus was measured by TCID_50_ assay daily, Days 1–3 post-challenge. Shedding kinetics for individual animals are shown in **a** (A/BOL13) and **c** (A/NC99). Each curve represents shedding data from an individual animal (four animals per dose group). Wt virus shedding was expressed as the geometric mean of virus titer over Days 1–3 post-challenge. Protection from wt virus shedding for monovlaent, trivalent, and quadrivalent LAIV groups is summarized in **b** (A/BOL13) and **d** (A/NC99). Data shown are geometric mean virus titers for individual animals (symbols), with four animals per dose group. Columns and error bars for all virus titer data show group median and interquartile range. Statistical comparison between groups was performed by two-way analysis of variance with Sidak’s post-test correcting for multiple comparisons. Assessment of protection from wt shedding compared vaccinated groups to unvaccinated controls. *P < 0.05, **P < 0.01, ***P < 0.001, ****P < 0.0001. Dotted lines indicate lower limit of detection; values below this were plotted as 0.5× the lower limit of detection. A/BOL13 A/Bolivia/559/2013, A/NC99 A/New Caledonia/20/1999, FFU fluorescent focus units, GMT geometric mean titer, LAIV live attenuated influenza vaccine, NT nasal turbinate, RT-qPCR quantitative reverse transcription-polymerase chain reaction, TCID_50_ tissue culture infectious dose 50%, wt wild type.

To simulate patient sampling in test-negative VE studies, post-challenge wt virus shedding was assessed by nasal swabs taken daily on Days 1–3 post-challenge, with virus titers measured by TCID_50_ and RT-qPCR. Figure 5 shows wt shedding kinetics and geometric mean Days 1–3 shedding for statistical assessment of protection in vaccinated groups.

MLAIV A/BOL13 vaccination provided significant reduction in median wt shedding relative to unvaccinated controls at all doses (*P* < 0.0001 for all groups). However, again, individual animals in each of the 4.0 and 3.0 log_10_ FFU dose groups showed no response to vaccination, failing to reduce wt virus titer (Fig. 5a).

Similar to the results from assessment of fever, all A/BOL13 TLAIV- and QLAIV-vaccinated groups showed incomplete protection from challenge virus replication. Only a 6.0 log_10_ FFU TLAIV dose gave a significant reduction in wt virus shedding, relative to unvaccinated controls (*P* = 0.0015). Even in this case, median wt shedding remained in excess of 3.0 log_10_ TCID_50_/ml. By contrast, A/NC99 vaccination gave significant reductions in wt shedding in all vaccine formulations at all doses, with the lone exception of the 3.0 log_10_ FFU QLAIV group. This indicated that, in line with the loss of protection against fever already shown, A/BOL13 in TLAIV and QLAIV provided reduced protection against wt virus replication, relative to MLAIV, while A/NC99 was less affected.

While fever and wt shedding endpoints were intended to simulate clinical VE study format, ferret respiratory tissues were also harvested for assessment of wt virus titer by TCID_50_ at Day 3 post-challenge. Challenge virus titers in nasal turbinate tissues provided similar observations to wt virus shedding, with A/NC99 conferring reduction in virus titer at lower doses than A/BOL13 in all formulations (Supplementary Fig. 6a and 6d). Due to insufficient volume for a subset of tissue samples, TCID_50_ data could not be generated for 4.0 log_10_ FFU A/NC99 TLAIV and QLAIV.

In lung tissue of A/BOL13-vaccinated animals, wt virus was undetectable in MLAIV-vaccinated animals as well as the majority of 5.0 or 6.0 log_10_ FFU TLAIV- and QLAIV-vaccinated ferrets (Supplementary Fig. 6c). However, wt virus titers in lung tissues were generally low by TCID_50_, including in unvaccinated controls. For greater sensitivity, lung titers were also assessed by RT-qPCR (Supplementary Fig. 6b). By RT-qPCR, 5.0 and 6.0 log_10_ FFU TLAIV doses resulted in undetectable levels of wt vRNA in all animals, while two of four animals in both 4.0 and 6.0 log_10_ FFU QLAIV groups were similarly protected.

For A/NC99, by TCID_50_, wt virus was undetectable in lung tissues even in unvaccinated control animals (Supplementary Fig. 6f). However, two control animals did give positive RT-qPCR readings (Supplementary Fig. 6e). Together, these observations suggested that successful wt upper respiratory tract infection did not guarantee development of lower respiratory tract infection by Day 3 post-challenge and that sterilizing protection of the lungs may be readily observed in this study format. This suggests that reduction in lung virus titer may provide an unrealistic assessment of LAIV clinical performance.

### A/BOL13 is susceptible to inter-strain competition in multivalent formulations

**Fig. 6:**
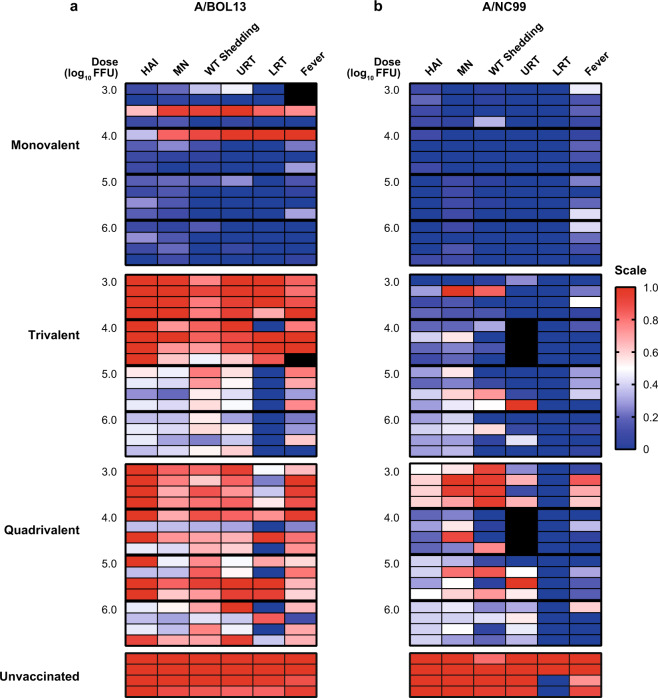
Summary of endpoints demonstrates reduction in A/BOL13 efficacy in multivalent formulations relative to A/NC99. Data from multiple endpoints for A/BOL13 **a** and A/NC99 **b** vaccine groups were expressed as heatmaps. Each panel shows a single vaccine formulation (top to bottom: monovalent, trivalent, and quadrivalent LAIV, and unvaccinated). Each row represents an individual ferret, with four animals per dose group, with vaccine doses ordered from 3.0 (top) to 6.0 log_10_ FFU (bottom). Endpoints shown are, left to right: HAI titer; MN titer; geometric mean wt virus shedding (Days 1–3, TCID_50_); wt virus titer in NT tissue (TCID_50_); wt virus titer in lung tissue (RT-qPCR); fever (°C). All endpoints, irrespective of units of measurement, were normalized to a uniform 0–1 scale. Immune responses and protection equivalent to the average of the 6.0 log_10_ FFU monovalent vaccine group were calibrated to 0 (navy blue). Immune responses and protection equivalent to average data from unvaccinated controls were calibrated to 1 (red). White cells indicate mean of red/navy blue. Gray cells indicate missing data points owing to insufficient tissue sample quantity or corrupt telemetry data. A/BOL13 A/Bolivia/559/2013, A/NC99 A/New Caledonia/20/1999, FFU fluorescent focus units, HAI hemagglutination inhibition, LRT lower respiratory tract, MN microneutralization, NT nasal turbinate, QLAIV quadrivalent LAIV, RT-qPCR quantitative reverse transcription-polymerase chain reaction, TCID_50_ tissue culture infectious dose 50%, URT upper respiratory tract, wt wild type.

Figure 6 shows data from individual animals across the various study endpoints as heatmaps. LAIV shedding was excluded from this analysis due to the low levels of detectable vRNA in low-dose groups and multivalent formulations. Following MLAIV vaccination, A/BOL13 proved effective in most animals across the dose range studied. However, a single animal in each of the 3.0 and 4.0 log_10_ FFU dose groups failed to respond to vaccination, with uniformly low responses and protection by all endpoints (Fig. 6a). By comparison, MLAIV A/NC99 was similarly effective across the entire dose range (Fig. 6b).

Incorporation of each H1N1 strain into otherwise equivalent TLAIV and QLAIV formulations resulted in striking differences in efficacy. TLAIV A/NC99 retained an almost MLAIV-like profile, with all animals responding positively to vaccination and subsequently protected from wt challenge. By contrast, A/BOL13 provided no protective efficacy in the 3.0 and 4.0 log_10_ FFU TLAIV-dose groups, with all animals comparable to unvaccinated controls. These heatmaps also highlighted a generalized reduction in magnitude for all A/BOL13 endpoints, other than lung virus titer, at 5.0 and 6.0 log_10_ FFU doses. These differences were further exacerbated in QLAIV-vaccinated animals. For A/NC99, only the 3.0 log_10_ FFU QLAIV dose group showed a clear reduction in effectiveness relative to MLAIV controls, confirming the benefit of its increased replicative fitness in multivalent formulations.

## Discussion

Hawksworth et al. previously showed that A/H1N1pdm09 LAIV strains suffered from reduced replicative fitness in hNEC, with A/H1N1pdm09 strain A/BOL13 producing peak viral titers approximately 100-fold lower than those of clinically effective, pre-2009 strain A/NC99^16^. Here, we further demonstrated that the reduced fitness of A/BOL13 was replicated in vivo, with similarly decreased levels of MLAIV shedding in ferrets relative to A/NC99. At low (3.0 and 4.0 log_10_ FFU) vaccine doses, animals vaccinated with MLAIV A/BOL13 began to lose protection, with 75% of animals protected at both doses, versus 100% for A/NC99. This small difference suggested that A/BOL13 infectivity in ferrets was reduced relative to A/NC99.

Selection of the A/BOL13 vaccine candidate was based on its properties for HA protein thermal stability and virus yield in eggs. A/BOL13 was naturally thermally stable and did not require any stabilizing mutations that might have affected virus replication^9^. However, to obtain sufficient egg yield, the A/BOL13 HA protein incorporated the residues N119 and D186, previously identified in an A/CA09 high growth reassortant^17^. However, both A/CA09 and A/BOL13 suffered from reduced replication in human nasal epithelial cells^16^. It is possible that these residues, while increasing egg yield, could have had a deleterious effect on virus replication in human cells and ferrets, although this has yet to be demonstrated.

Comparison of shedding by RT-qPCR (total virus) and TCID_50_ (infectious virus) suggested that MLAIV A/BOL13 generated significantly more non-infectious progeny than MLAIV A/NC99, wt A/BOL13 or wt A/NC99 (Supplementary Fig. 1). Earlier studies had demonstrated that multi-cycle infectivity (TCID_50_) for A/BOL13 in tissue culture was significantly lower than single-cycle (FFU) infectivity^16^. These observations suggested that the reduced fitness of A/BOL13 was at least partly due to a deficiency in fully infectious virus production.

Gould et al. hypothesized that the high content of defective interfering (DI) influenza A observed in 2014–15 QLAIV FluMist sprayers may have contributed to the suboptimal 2013−14A/CA09 VE^18^. DI particles are spontaneously generated mutated viruses containing significant internal gene segment deletions, which can interfere with virus replication. Theoretically, high levels of DI viruses in a vaccine strain could reduce viral fitness and immunogenicity. However, more recent work suggests that DI viruses are not associated with low A/H1N1pdm09 fitness or VE^19^, and that A/BOL13 possessed a non-DI-based deficiency in infectious virus production.

Recently, Dadonaite et al. demonstrated the importance of inter-segment interactions in efficient influenza virus genome assembly^20^. It is possible that the HA and neuraminidase (NA) gene segments of A/BOL13 might assemble less efficiently with the heterologous internal genes of A/Ann Arbor/06/60 master donor virus than the homologous internal genes of wt A/BOL13, leading to an excess of non-infectious particles. Understanding the mechanism behind reduced LAIV fitness remains an important question, and the effect of non-infectious virus content on VE continues to be an area of active study for our group.

The incorporation of A/BOL13 into TLAIV and QLAIV vaccine formulations considerably reduced its in vivo efficacy relative to MLAIV. By contrast, fitter A/NC99 retained the ability to confer relatively greater protection in TLAIV and QLAIV. At study outset, our hypothesis was that a less fit virus subject to competition in multivalent formulations would require a higher vaccine dose to be efficacious. Cherukuri et al. had also shown that historic LAIV formulations remained immunogenic at doses lower than the 7.0 log_10_ FFU human dose typically used in ferrets^21^. The dose range applied here was thus intended to reveal lower dose efficacy for the fitter A/NC99, and a requirement for a higher protective dose for A/BOL13. Consequently, a 7.0 log10 FFU dose group was not included in the study in an effort to reduce animal numbers.

The hypothesis was confirmed, as the minimum protective QLAIV dose for A/NC99 against both wt shedding and fever was 4.0 log_10_ FFU, while for A/BOL13 this was not achieved even at the highest 6.0 log_10_ FFU dose. This wide disparity in QLAIV efficacy showed that A/BOL13 was more susceptible to inter-strain competition than A/NC99. Furthermore, this showed that the reduced A/BOL13 QLAIV VE seen in pediatric clinical studies^5^ could be reproduced most effectively using low, multivalent vaccine doses. The 4.0 log_10_ FFU dose has since been selected as providing the greatest distinction between strains of differing fitness and applied to all ongoing work with this model. These data also confirmed that the clinically relevant endpoints of fever and wt shedding could describe the ability of H1N1 strains to induce serum immune responses and protect animals from wt virus challenge.

The potential value of low-dose vaccination for describing inter-strain competition was seen in a recent study with an A/Leningrad/17/57 (LEN57)-based A/CA09 LAIV^22^. In this study, the A/H1N1pdm09 strain A/CA09 showed minimal evidence of competition in TLAIV or QLAIV in ferrets, conferring significant protection from challenge in both formulations^22^. However, LEN57 A/CA09 demonstrated variable efficacy, from zero to moderate, in TLAIV clinical trials^23,24^. While multiple factors likely contributed to this difference, the high vaccine dose (7.0 log_10_ 50% egg infectious dose [EID_50_] for A strains, 6.5 log_10_ EID_50_ for B strains) used in this study may have precluded clinically relevant assessment of competition or efficacy in the ferret model^22^.

Observations of varying efficacy between doses and formulations emphasize the need for accurate and comparable quantification of vaccine preparations. Here, vaccine formulations were titrated by fluorescent focus assay (FFA; see “Methods”), rather than TCID_50_ or EID_50_. FFA provides a rapid approach that can detect multiple virus subtypes with strain-specific antibodies, irrespective of valency. Titration of multivalent vaccines by TCID_50_ or EID_50_ requires neutralization of subtypes not being measured, prior to infection of cells or eggs, introducing potential variability. It should be noted that the vaccine material used in these studies was not commercially manufactured or titrated. As such, vaccine virus titers may have been more prone to variability than those used in clinical applications. This could have contributed to the inconsistent dose-response effect seen for A/BOL13 shedding at a 4.0 log_10_ FFU dose, where shedding in TLAIV was significantly lower than QLAIV. However, as these vaccine virus shedding differences did not correlate with protection endpoints, it is also possible that variations in nasal swab sampling and RT-qPCR measurement of shedding led to this observation.

Another caveat to quantification by FFA was described in Hawksworth et al. where less fit H1N1 LAIV strains possessed lower TCID_50_ than FFA titers^16^. This indicated reduced multi-cycle infectivity in less fit strains and correlates with the observation of increased non-infectious MLAIV A/BOL13 shedding in ferrets. Consequently, any future vaccine candidates are now expected to show comparable TCID_50_ and FFA titers prior to incorporation into vaccine formulations.

Having demonstrated the in vivo impact of reduced H1N1 fitness, the assessment of vaccine candidate replication in hNECs, along with comparison of multi-cycle versus single-cycle infectivity, is now integral to the strain selection process. Together these assessments are expected to reduce the likelihood of selection of low VE strains. Data correlating precise replicative properties of novel strains with efficacy in ferrets also continue to be gathered.

While the primary goal of the work presented was to describe the effect of competition on H1N1 strains, reduced H3N2 A/SWITZ13 shedding and serum immune responses were also observed upon incorporation of A/NC99 into the 2015–16 QLAIV in place of A/BOL13. This suggested that competition could affect LAIV subtypes other than H1N1, although the clinical relevance of this is not yet clear.

In a recent pediatric shedding and immunogenicity study, A/SWITZ13 in the 2015−16A/BOL13 QLAIV was shown to shed virus and generate HAI responses similarly to highly effective H3N2 strains^25^. This supported the detection of shedding and HAI responses in the same formulation presented here. However, minimal VE data for A/SWITZ13 in 2015–16 were available^26^, making correlation with VE difficult. There are no clinical or ferret efficacy data for A/SWITZ13 formulated with A/NC99 and it is unknown whether the reduction in A/SWITZ13 shedding and immunogenicity seen in A/NC99 QLAIV-vaccinated ferrets would have affected efficacy. Consequently, studies are being extended to describe the association of LAIV shedding and seroconversion with efficacy for both H3N2 and B viruses in ferrets.

While the data presented demonstrate clear differences in A/BOL13 and A/NC99 efficacy in multivalent vaccines, there are potential caveats to the comparison of pre-2009 H1N1 and A/H1N1pdm09 strains. A/BOL13 wt challenge produced higher levels of fever and wt virus shedding than wt A/NC99. Different stringencies of wt challenge could have potentially influenced the relative efficacy of these strains. However, the fact that the reduced protection from challenge provided by A/BOL13 was mirrored in pre-challenge endpoints, mitigates this concern. Nonetheless, reproducing differences in VE between A/H1N1pdm09 strains with varying fitness and more similar wt challenge remains a significant goal and a subject of continuing research.

Another key feature distinguishing our ferret model from clinical investigations, is that all work was performed in influenza-naive ferrets, while clinical VE data are generated from real-world populations with diverse ages and infection histories. In humans, frequencies of seroconversion and LAIV shedding decrease with increasing age or prior vaccination^25,27,28^ and an individual’s pre-existing immune landscape can influence the antibodies generated in response to influenza vaccination^29,30^. As A/BOL13 in QLAIV formulation generated variable clinical VE data^5,8^, we took a consensus view to allow evaluation in vivo, i.e. A/BOL13 in QLAIV should give lower efficacy than A/NC99 in ferrets. Reassuringly, the data presented support this generalization. However, our model might most accurately represent vaccination in young, seronegative children receiving a single dose of vaccine, as undertaken in the UK. Thus, it will be of great interest to examine the effects of prior infection or vaccination, or receipt of two doses of vaccine, on LAIV clinical outcomes using this model.

This body of work has identified a number of important findings for LAIV strain selection. First, we have demonstrated that the reduced fitness of A/BOL13 rendered it more susceptible to inter-strain competition in TLAIV and QLAIV formulations than the clinically effective pre-2009 H1N1 strain A/NC99, identifying and demonstrating the likely root cause for the reduced clinical VE of A/BOL13 seen in multivalent vaccine formulations. Our findings also validate the use of fever and wt shedding as clinically relevant endpoints that can be used to measure the ability of H1N1 strains to induce serum immune responses and protect animals from wt virus challenge. Based on the understanding generated by this work and the selection of an H1N1 strain with greater replicative fitness (A/Slovenia/2903/2015), ACIP reinstated the recommendation for LAIV as an influenza vaccine option for indicated persons in the 2018−19 season^10^. Future work will be useful to further validate the clinical translatability of this model in real-world populations with diverse ages and influenza infection/vaccination histories.

More broadly, the identification of inter-strain competition in the context of less fit vaccine strains highlights this as a factor to consider when developing other multivalent vaccines. Finally, defining a low, ferret-appropriate vaccine dose for LAIV reveals a key role for dose optimization and the use of ferrets as an animal model in broader pre-clinical vaccine development.

## Methods

### Cells and viruses

All vaccine formulations described were generated from non-commercial material for use in the studies. Madin–Darby canine kidney (MDCK) cells, used in FFA, TCID_5_, and MN assays, were obtained from the American Type Culture Collection (ATCC) and passaged fewer than 20 times prior to use in analytical tests. No other cell line authentication was conducted. MDCK cells were cultured and maintained in Eagle’s minimum essential medium with non-essential amino acids at 37 °C and 5% CO_2_, as previously described^16^.

LAIV viruses were propagated in the allantoic cavity of 10–11-day-old embryonated hens’ eggs at 33 °C. All were 6:2 reassortants carrying the six internal gene segments (PB2, PB1, PA, NP, M, and NS) of cold-adapted A/AnnArbor/6/1960^31^ or cold-adapted B/AnnArbor/1/1966^32^, and the HA and NA gene segments of the following viruses: Pre-2009 H1N1 A/New Caledonia/20/1999 (A/NC99). A/H1N1pdm09—A/Bolivia/559/2013 (A/BOL13). H3N2—A/Perth/16/2009 (A/PER09); A/Switzerland/9715293/2013 (A/SWITZ13). B/Yamagata – B/Phuket/3073/2013 (B/PHUK13). B/Victoria—B/Brisbane/60/2008 (B/BRIS08). Wt viruses were propagated from egg-derived wt as for vaccine viruses. Viruses were titrated by fluorescent focus assay (FFA) as described in “Virus quantification” of the “Methods” section^33^.

### Ferrets

Animal testing performed by AstraZeneca/MedImmune follows requirements set out by the Home Office under the Animal Scientific Procedures Act 1986 and all work is performed under an appropriate project license. Ethical approval of the study was provided by AstraZeneca’s Council for Science & Animal Welfare (C-SAW) prior to study start. Work described here was conducted at Charles River Laboratories (CRL) Ireland Ltd, Carrentrila, Ballina, Co. Mayo, Ireland. F26 D786, in compliance with the European Directive for the Protection of Animals used for Scientific Purposes, Directive 2010\63\EU, as transposed into Irish law under Statutory Instrument, S.I. No. 543 of 2012 (as amended). Charles River Laboratories Ireland Ltd holds a Health Products Regulatory Authority Project authorization to carry out influenza testing in ferrets (AE19108/P022). This work was conducted under this authorization. The Project Manager holds a valid individual authorization from the HPRA (No. 19108\I079). All staff who performed procedures and euthanasia as part of this work were trained and hold valid HPRA individual authorizations to perform these tasks.

Studies were conducted in outbred, mixed sex, specific pathogen-free, 14–26-week-old ferrets (Mustela putorius furo; Charles River Laboratories Ltd, Ballina, Ireland). Ferrets were confirmed seronegative for circulating H1N1, H3N2, and B viruses by HAI assay and randomly assigned to study groups. Animals were then housed in pairs with environmental enrichment. Each ferret was subjected to a health check on arrival at Charles River Laboratories and then daily during acclimatization, including twice-daily body temperature recordings. Post-inoculation, any adverse health observations were reported to and investigated by the designated veterinarian.

### Ferret studies

At least 10 days prior to vaccination, intraperitoneal telemetry chips (Data Sciences International, St. Paul, Minnesota, USA: cat.ANIPILL 0.1C) were surgically implanted. Body temperatures were then remotely monitored hourly (Data Sciences International: cat.Aniview V2), from pre-vaccination to study termination.

On Study Day 0, groups of four ferrets were each lightly sedated with isoflurane (C&M Vetlink, Annacotty, Ireland: cat.OVIV030) and intranasally vaccinated with a single 0.2 ml (~0.1 ml/nare) dose. Groups received either mock vaccination (phosphate-buffered saline [PBS; ThermoFisher Scientific, Waltham, Massachusetts, USA] with 1× sucrose phosphate [ThermoFisher Scientific: custom product, cat.AC10210390] and 1× gelatine-arginine-glutamate [ThermoFisher Scientific: custom product, cat.AC10207676]), or LAIV in MLAIV, TLAIV, or QLAIV formulations, at doses of one of 3.0, 4.0, 5.0, or 6.0 log_10_ FFU/strain/animal, in the same sample diluent. FFA titers for all strains were required to be within 0.2 log_10_ FFU of the targeted dose.

To evaluate LAIV virus shedding following vaccination, nasal swab samples were taken daily on Days 1–5 post-vaccination. Animals were anesthetized by intramuscular injection of 0.1 mg/kg Medetor (medetomidine; Chanelle Veterinary, Loughrea, Co Galway, Ireland: cat.PH003) before sedation with isoflurane. A Copan FloqSwab (Copan, Murrieta, California, USA: MINI UTM [Universal Transport Medium]) kit 1 ml (medium plus pernasal flocked swab, cat.360C) was inserted into the right nostril, rotated to ensure full contact with the mucosal surface, eluted by light vortexing in 1 ml of Copan universal transport medium and then aliquoted and stored at –80 °C. Medetor effects were reversed by intramuscular administration of 0.1 mg/kg Revertor (Chanelle Veterinary: cat.PH005), at least 30 min after the sedative.

On Days 7, 14, 21, and 27 post-vaccination, blood samples were taken for analysis of serum immune responses. Animals were sedated with isoflurane as above and 2 ml bleeds were taken from the superior vena cava. Blood samples were transferred to BD Vacutainer SST tubes (Aquilant Ltd, Dublin, Ireland: cat.366882). Coagulation could proceed for 30–90 min. Tubes were then centrifuged and serum decanted to cryovials for storage at –80 °C.

On Day 28 post-vaccination, animals were lightly sedated with isoflurane, as per vaccination, and intranasally inoculated with 5.0 log_10_ FFU of wt challenge virus in a 0.2 ml dose (~0.1 ml/nare). Nasal swab samples for wt shedding were then taken on Days 1–3 post-challenge, as for LAIV shedding above. On Day 3 post-challenge, ferrets were then euthanized by intracardiac injection of 1.5 ml of sodium pentobarbitone (200 mg/ml; Chanelle Veterinary: cats.MB0022, GA0022), and the lower left lobe of the lung and nasal turbinate tissues were harvested by standard methods. Tissues were ground with sterile pestle and mortar before adding 10× volume (v/w) of 2× Eagle’s medium, giving ~10% weight/volume suspension. Tissue suspensions were centrifuged and the 10^–1^ supernatant transferred to cryovials and stored at –80 °C. Telemetry chips were recovered from the intraperitoneal cavity.

### Quantification of fever

The initial factor influencing clinical test-negative VE study data is the self-reporting of ILI. To increase the clinical translatability of the ferret model, a quantifiable measure of ILI was required. To achieve this, intraperitoneal telemetry monitors were used to record core body temperatures for individual study animals hourly from time of vaccination to termination. Animal handling records identified all subsequent inoculation or sampling intervention times requiring anesthesia; corresponding hypothermic dips in body temperature were evident in pooled study data (Supplementary Fig. 7a). The period of 2 h pre-anesthetic to 10 h post-anesthetic was defined as ‘anesthetized’, representing the mean time between a short pre-anesthetic body temperature increase (due to animal handling) and a post-hypothermia return to normal temperature. These data were considered artefactual and were removed to enable accurate assessment of fever (Supplementary Fig. 7b). Post-challenge temperature profiles of individual animals were reconstructed using splines (fitted lines comprised of several quadratic curves) in JMP v13.0 statistical analysis software (Supplementary Fig. 7c). A spline ‘stiffness’ variable (lambda) of 5 × 10^11^ was selected to produce a root mean square error between 0.2 and 0.5 °C for most animals, based on the expected variability of the temperature measurements. The censored data were then replaced with the spline values, creating an interpolated series that approximated the body temperature expected if the animals had not been anesthetized.

The average temperature profile of each unvaccinated control group (four animals) was then used to define the post-challenge ‘fever period’ for each wt virus. First, control animals’ time stamps were rounded to the nearest hour and an average hourly body temperature calculated. The fever period start was then defined as the first of three post-challenge time-points where the control group’s average body temperature was >1.5 standard deviations above its average pre-challenge baseline body temperature. Fever period end was defined as the first of three group average temperature measurements returning below the same limit, or study end if this occurred first.

Hourly delta-temperature values were calculated for all animals during the fever period, subtracting the animal’s baseline body temperature from each fever period value. A single ‘fever’ value was calculated per animal using the average of all delta-temperature values during the fever period. This represented, for each animal, the average deviation from normal body temperature for the period that the wt challenge virus caused fever in unvaccinated controls.

### Virus quantification: fluorescent focus assay

Titration of MLAIV, TLAIV, and QLAIV formulations, and wt influenza challenge virus stocks, was performed by immunofluorescent staining of infected MDCK cells. Confluent MDCK cells in 96-well tissue culture plates were washed twice in FFA virus growth medium (FVGM). FVGM was prepared from Eagle’s minimal essential medium (EMEM; Lonza, Basel, Switzerland, cat.12-662Q) with 50 µg/ml gentamicin sulfate (Life Technologies, Carlsbad, CA, USA, cat.15750-078), 2 mM L-glutamine (Sigma, cat.25030081), and 0.5 µg/ml amphotericin B (Life Technologies, cat.15290018). Washed cells were then inoculated with 100 µl of 1:3 serial dilutions of vaccine preparations in FVGM. Duplicate plates were prepared for titration of each relevant subtype. Infected plates were then incubated at 33 °C for 18–20 h, in the absence of trypsin.

Following incubation, cells were washed with FVGM and fixed with 80% acetone (VWR, Radnor, PA, USA, cat.100033P) in water, for 20 min–2 h at –20 °C. Fixed plates were dried at 36 °C for 30 min and then washed with PBS containing 0.05% Tween-20 (PBST, Sigma-Aldrich, St. Louis, Missouri, USA, cat.P-3563). Plates were then incubated at 36 °C for 1 h with subtype-specific, anti-HA protein primary antibodies, either obtained from the National Institute for Biological Standards and Control, UK (NIBSC), or produced internally at AstraZeneca. Primary antibodies were diluted in PBST with 1% bovine serum albumin (BSA; Sigma, cat.A-2153) and used to detect the strains described in these studies as follows: A/NC99—sheep anti-A/Brisbane/59/2007 (NIBSC, Lot: 10/120); A/BOL13—sheep anti-A/CA09 (NIBSC, Lot: 14/130); A/SWITZ13—sheep anti-A/Hong Kong/4801/2014 (NIBSC, Lot: 15/236); B/PHUK13—mouse anti-B/Yamagata, SP12-051 (AstraZeneca); B/BRIS08—mouse anti-B/Victoria, C14975 (AstraZeneca). Cells were then washed twice with PBST and stained for 1 h at 36 °C with fluorescent secondary antibodies diluted in PBST with 1% BSA: A strains—alexa 488 donkey anti-sheep IgG (H&L) (Life Technologies, cat.A11015), B strains—alexa 488 goat anti-mouse (Life Technologies, cat.A11017).

Stained plates were washed twice with PBST followed by a single wash with sterile water. Washed plates were dried at room temperature for 30 min, and then fluorescent foci were counted using an Eclipse Ti inverted fluorescence microscope (Nikon, Tokyo, Japan) at ×100 magnification. Foci were counted in four infected wells for each strain; consisting of two consecutive dilutions on duplicate plates, each containing between 15 and 200 foci. The concentration of virus in the original vaccine formulation was then calculated.

### Virus quantification: TCID_50_

Quantification of monovalent LAIV and wt viruses in nasal swab samples was performed by TCID_50_ assay as a measure of fully infectious virus, as previously described^16^. MDCK cells in 96-well tissue culture plates were inoculated with 1:10 dilutions of nasal wash samples in the presence of 1:400 10xTrypLE (Life Technologies; Carlsbad, California, USA). Infected cells were incubated at 33 °C for 6 days and scored for cytopathic effect on an inverted light microscope. TCID_50_ titers were determined by the Spearman–Karber method^34,35^.

### RNA extraction and multiplex RT-qPCR

Extraction of RNA from nasal swab samples was performed on the QIAcube HT automated platform, using the QIAamp 96 Virus QIAcube HT kit (Qiagen, Hilden, Germany: cat.57731), according to manufacturer’s instructions. RNA samples were eluted in 100 µl of buffer AVE and stored at –80 °C.

To compare LAIV shedding between MLAIV, TLAIV, and QLAIV formulations in nasal swabs, a multiplex RT-qPCR assay was developed, with each vaccine subtype detected via its HA (BVic) or NA (H1N1, H3N2, BYam) gene segment. A total of 37 influenza A and 12 influenza B strain HA and NA sequences between 2009 and 2016 were obtained from the GISAID EpiFlu™ database (strains listed in Supplementary Table 1) and aligned using MegAlign Software (DNASTAR) to identify conserved regions exclusive to each subtype. These regions were targeted to design subtype-specific primer and probe sets (Supplementary Table 2). Primer and probe designs were tested for efficiency (efficiency of 90–110% accepted), screened for cross reactivity, and multiplexed to enable simultaneous detection of all four subtypes. Reference gene glyceraldehyde-3-phosphate dehydrogenase primers and probes, described by Carolan et al.^36^, were then selected for sample normalization following an assessment of precision and sensitivity.

For quantification of viral copy number, RNA reference standards were generated. RNA was extracted from LAIV material using the QIAamp 96 Virus QIAcube HT kit (Qiagen, cat#: 57731). One-step reverse transcription-polymerase chain reaction was performed using the SuperScript III one-step kit (ThermoFisher, cat#: 12574-026) incorporating T7 promoter sites to enable synthesis of negative-sense vRNA. The polymerase chain reaction product was purified using the Qiaquick purification kit (Qiagen kit, cat.28106) and RNA was in vitro T7 transcribed and deoxyribonuclease treated using the MEGAscript T7 kit (ThermoFisher, cat.AM1334). T7 RNA was purified using the MEGAclear kit (ThermoFisher, cat.AM1908). RNA was quantified using the Quant-iT kit (ThermoFisher, cat.Q33140). RNA reference standards were then diluted in RNA storage buffer (ThermoFisher, cat.AM7001) to 2 × 10^8^ copies/µl and serially diluted 10-fold to 2 × 10^2^ copies to generate a standard curve. All kits used in the synthesis of RNA reference standards were used according to manufacturer’s instructions. All primer and probe sequences are detailed in Table S2.

Reverse transcription qPCR reactions consisted of 1× TaqPath™ 1-Step Multiplex Master Mix (ThermoFisher, cat.A28526), 250 nM of primers/probe, plus 12 µl of extracted ferret nasal swab RNA, and were made up to a total reaction volume of 25 µl with nuclease-free water (ThermoFisher, cat.10977035). Reverse transcription qPCR was performed in a one-step reaction using the QuantStudio™ 5–96-well 0.2 ml block. Cycling conditions were 25 °C for 2 min, 53 °C for 10 min, 95 °C for 2 min, followed by 40 cycles of 95 °C for 15 s and 60 °C for 1 min. Data were gathered and analyzed with the ThermoFisher Connect™ qPCR Design and Analysis Application. vRNA copies/mL was determined by comparison with a 10-fold serially diluted RNA reference standard of known concentration included on each 96-well plate.

MLAIV infectious virus shedding was measured by TCID_50_ and RT-qPCR, while only RT-qPCR was used for comparison of shedding in MLAIV, TLAIV, and QLAIV formulations.

### Serum immune responses: HAI

HAI assays were performed with standard methodology. Ferret antiserum (100 µl) was added to 150 µl 2× receptor-destroying enzyme (Deben Diagnostics, Ipswich, UK: cat.370013) and incubated at 37 °C for 18–20 h. Sodium citrate (150 µl of 2% w/v; Sigma-Aldrich, St. Louis, Missouri, USA: cat.W302600) was then added and the reaction was heat inactivated at 56 °C for 45 min. Treated antiserum was serially diluted 1:2 and added to an equal volume of virus (8HAU/well). Virus/antibody complexes were incubated at room temperature for 30–40 min before addition of one-well volume of 0.5% chicken (H1N1 and B) or guinea pig (H3N2) red blood cell suspension. Virus/antibody/red blood cell mix was incubated at room temperature for 60 min and HAI endpoints recorded as the reciprocal of the highest dilution of antiserum able to fully prevent agglutination. If no endpoint was detectable, then a reading of 2 was recorded (1/2 lower limit of detection), to enable statistical analysis.

### Serum immune responses: MN

Serially 1:2 diluted, receptor-destroying enzyme-treated antisera were generated in 96-well plates as per HAI. An equal volume of target virus (concentration 2000 TCID_50_/ml) was added per well, then incubated at room temperature for 1 h. Virus–antibody complex (50 µl; final virus concentration: 100 TCID_50_/well, minimum serum dilution: 1:8) was then transferred to washed 96-well plates of MDCK cells. Infected cells were incubated for 6 days at 33 °C and cytopathic effect measured. Neutralization endpoints were calculated as the reciprocal of the highest antiserum dilution able to completely neutralize virus infection. Duplicate assays were conducted, and neutralization titers were calculated as the geometric mean of duplicate endpoints. If no endpoint was detectable then a reading of 4 was recorded (1/2 lower limit of detection), to enable statistical analysis.

### Statistical analysis

Analyses are described in the figure legend. All graphs were generated in GraphPad Prism, version 8.0 (GraphPad Software Inc, San Diego, CA, USA). Statistical analyses were performed in JMP v14 (JMP Software, SAS Institute Inc, Marlow, Bucks, UK).

### Reporting summary

Further information on research design is available in the Nature Research Reporting Summary linked to this article.

## Supplementary information

Supplementary Information

Reporting Summary

## Data Availability

All data associated with this study are available in the main text or the supplementary materials. Further information and requests for resources and reagents should be directed to and will be fulfilled by the Lead Contact, Oliver Dibben (oliver.dibben@astrazeneca.com).
