# Supplementary material for: Defining the root cause of reduced H1N1 live attenuated influenza vaccine effectiveness: low viral fitness leads to inter-strain competition
Source: NPJ Vaccines. 2021 Mar 12;6:35. doi: 10.1038/s41541-021-00300-z (PMC7955111; doi:10.1038/s41541-021-00300-z)
Supplement: Supplementary file 1 — Supplementary Information [file 41541_2021_300_MOESM1_ESM.pdf]

LIST OF SUPPLEMENTARY MATERIALS

Supplementary Table 1. Influenza strains aligned for design of quantitative reverse transcription polymerase chain reaction primers and probes to subtype-specific conserved regions.

Supplementary Table 2. Primers and probes for RT-qPCR analysis.

Supplementary Fig. 1. Assessment of non-infectious particle shedding by H1N1 strains.

Supplementary Fig. 2. H1N1 LAIV shedding kinetics in individual animals.

Supplementary Fig. 3. H3N2 LAIV shedding kinetics in individual animals.

Supplementary Fig. 4. B LAIV shedding kinetics in individual animals.

Supplementary Fig. 5. Serum MN responses to vaccination.

Supplementary Fig. 6. A/BOL13 provides reduced protection against wt challenge virus replication in respiratory tissues.

Supplementary Fig. 7. Quantitative analysis of ferret fever development.

SUPPLEMENTARY MATERIALS

Supplementary Table 1. Influenza strains aligned for design of quantitative reverse transcription polymerase chain reaction primers and probes to subtype-specific conserved regions

Year of Isolation	Strain	Subtype
2009	A/California/07/2009	H1N1
	A/Perth/16/2009	H3N2
	A/Victoria/210/2009	H3N2
	A/Hong Kong/26560/2009	H3N2
2010	A/Brisbane/10/2010	H1N1
	A/Perth/10/2010	H3N2
	A/Iowa/19/2010	H3N2
	B/Wisconsin/01/2010	B-Yam
2011	A/Christchurch/28/2011	H3N2
	A/Victoria/361/2011	H3N2
	A/Berlin/93/2011	H3N2
	B/Nevada/03/2011	B-Vic
	B/Texas/06/2011	B-Yam
2012	A/Hawaii/22/2012	H3N2
	A/Texas/50/2012	H3N2
	A/Ohio/2/2012	H3N2
	A/New York/39/2012	H3N2
	B/Massachusetts/02/2012	B-Yam
	A/Washington/24/2012	H1N1
2013	A/Dominican Rep/7293/2013	H1N1
	A/Bolivia/559/2013	H1N1
	A/New Hampshire/04/2013	H1N1
	A/South Africa/3626/2013	H1N1
	A/American Samoa/4786/2013	H3N2

	A/Costa Rica/4700/2013	H3N2
	A/Switzerland/9715293/2013	H3N2
	B/Texas/2/2013	B-Vic
	B/Phuket/3073/2013	B-Yam
2014	A/Palau/6759/2014	H3N2
	A/Hong Kong/4801/2014	H3N2
	A/New Caledonia/71/2014	H3N2
	A/Laos/1187/2014	H1N1
	A/New Caledonia/58/2014	H1N1
	A/Tasmania/24/2014	H1N1
	B/Hawaii/24/2014	B-Vic
2015	A/South Australia/9/2015	H3N2
	A/Alaska/232/2015	H3N2
	A/Michigan/45/2015	H1N1
	A/St Petersburg/61/2015	H1N1
	A/Lisboa/32/2015	H1N1
	A/Singapore/GP1908/2015	H1N1
	A/Shanghai-Putuo/1860/2015	H1N1
	A/Slovenia/2903/2015	H1N1
	B/Indiana/25/2015	B-Vic
	B/Florida/78/2015	B-Vic
	B/Maryland/12/2015	B-Yam
	B/California/12/2015	B-Yam
	B/Arizona/10/2015	B-Yam
2016	A/Idaho/33/2016	H3N2

Supplementary Table 2. Primers and probes for RT-qPCR analysis

Purpose	Gene Target	Subtype	Seq 5'-3'	Manufacturer
Reference standard synthesis	NA	H1N1 & H3N2	***TAATACGACTCACTATAGGG****AGTAGAAACAAGGAGTTTTT*[1]	IDT
		B Yamagata	***TAATACGACTCACTATAGGG****AGTAGTAACAAGAGCATTTTTCAG* *	IDT
	HA	B Victoria	***TAATACGACTCACTATAGGG****TAGTAGTAACAAGAGCATTTTTC*[1]	IDT
RT-qPCR	NA	H1N1 Pre-Pandemic	*TACTAAATCAATAGAGTTGAATGCACC*	IDT
			ACTGGATTACAGCTGCCCTCTCC	IDT
			FAM-TCAAATAGGATACATCTGCAGTGGG-MGBNFQ	ThermoFisher
		H1N1 Post-Pandemic	*AGTCAAATCAGTCGAAATGAAAGCC*	IDT
			TACTGGACCACAACTGCCTGTCT	IDT
			FAM-TCAGATGGGATACATATGCAGTGGG-MGBNFQ	ThermoFisher
		H3N2	*CGTTCATACTAGCACATTGTCAGGA*	IDT
			CCAAACAATGGCTACTGCTGGAGC	IDT
			VIC-TGTTTCCAGTTATGTGTGTTCAGGA-MGBNFQ	ThermoFisher
		B Yamagata	*CAAGATTGGAAGGTGGTACTCTCG*	IDT
			TCTATCCCAATACAGGGGACATC	IDT
			*ROX-AC**T**GACAG**T**GAAGCCC**TT**GCTCT-Iowa black 3IAbRQSp*[2]	IDT
	HA	B Victoria	*GGAGGTCAATGTGACTGGTGT*	IDT
			TTTTGTTCTGTCGTGCATTATAGG	IDT
			NED-TGGGCAGACCAAAATGCAC-MGBNFQ	ThermoFisher
Sample normalization	GAPDH	Not applicable	*TGCGGCCAAGGCAGTAG*	IDT
			AGGCCATGCCAGTGAGCTT	IDT
			VIC-CTGAGCTGAATGGGAAG-MGBNFQ	IDT

Primer and probe sequences outside the date ranges of 2009–2016, as described in Materials and Methods: RNA extraction and multiplex RT-qPCR, are underlined for each RT-qPCR primer/probe set. [1]T7 sequence highlighted in bold text, [2]Super T bases highlighted in bold text.
GAPDH, glyceraldehyde-3-phosphate dehydrogenase; HA, hemagglutinin; IDT, Integrated Device Technology, Inc.; NA, neuraminidase; RT-qPCR, quantitative reverse transcription polymerase chain reaction.

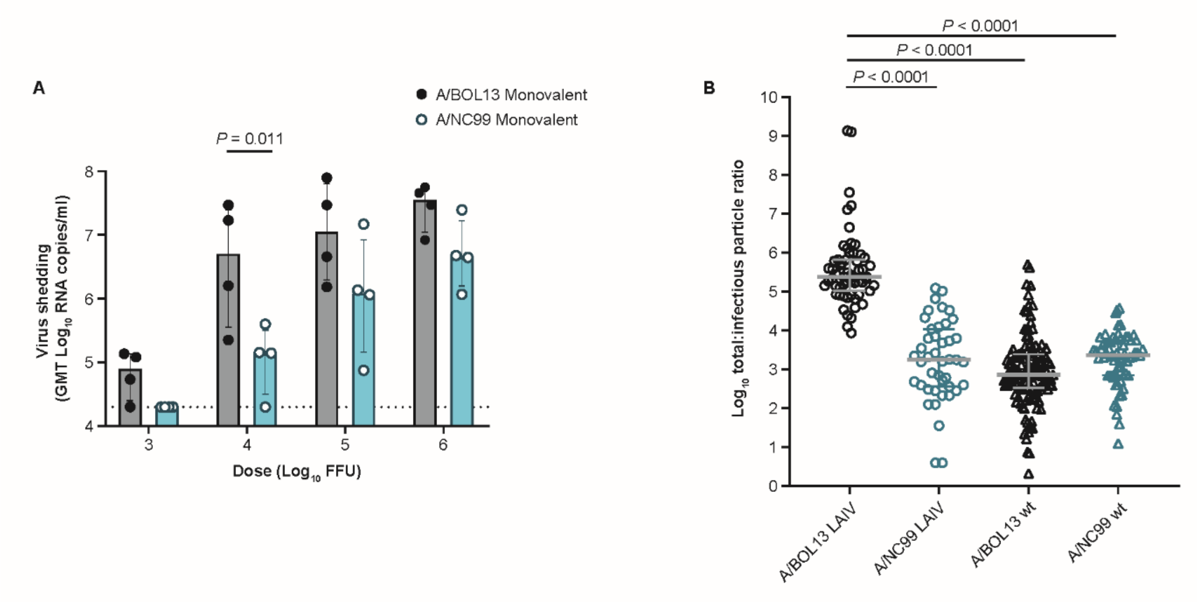

Supplementary Fig. 1. Assessment of non-infectious particle shedding by H1N1 strains. a A/BO13 versus A/NC99 monovalent LAIV geometric mean Days 1–5 shedding measured by RT-qPCR. Columns and error bars show median and interquartile range of four animal groups. **b** Ratio of monovalent LAIV and wt total virus (RT-qPCR copy number) to infectious virus ($TCID_{50}$) shedding. Symbols represent individual nasal swab samples from Days 1–5 post-monovalent LAIV vaccination or Days 1–3 post-wt challenge. Dotted lines indicate lower limit of detection; values below this were plotted as 0.5x the lower limit of detection. A/BOL13, A/Bolivia/559/2013; A/NC99, A/New Caledonia/20/1999; FFU, fluorescent focus units; GMT, geometric mean titer; LAIV, live attenuated influenza vaccine; RT-qPCR, quantitative reverse transcription polymerase chain reaction; $TCID_{50}$, tissue culture infectious dose 50%; wt, wild-type.

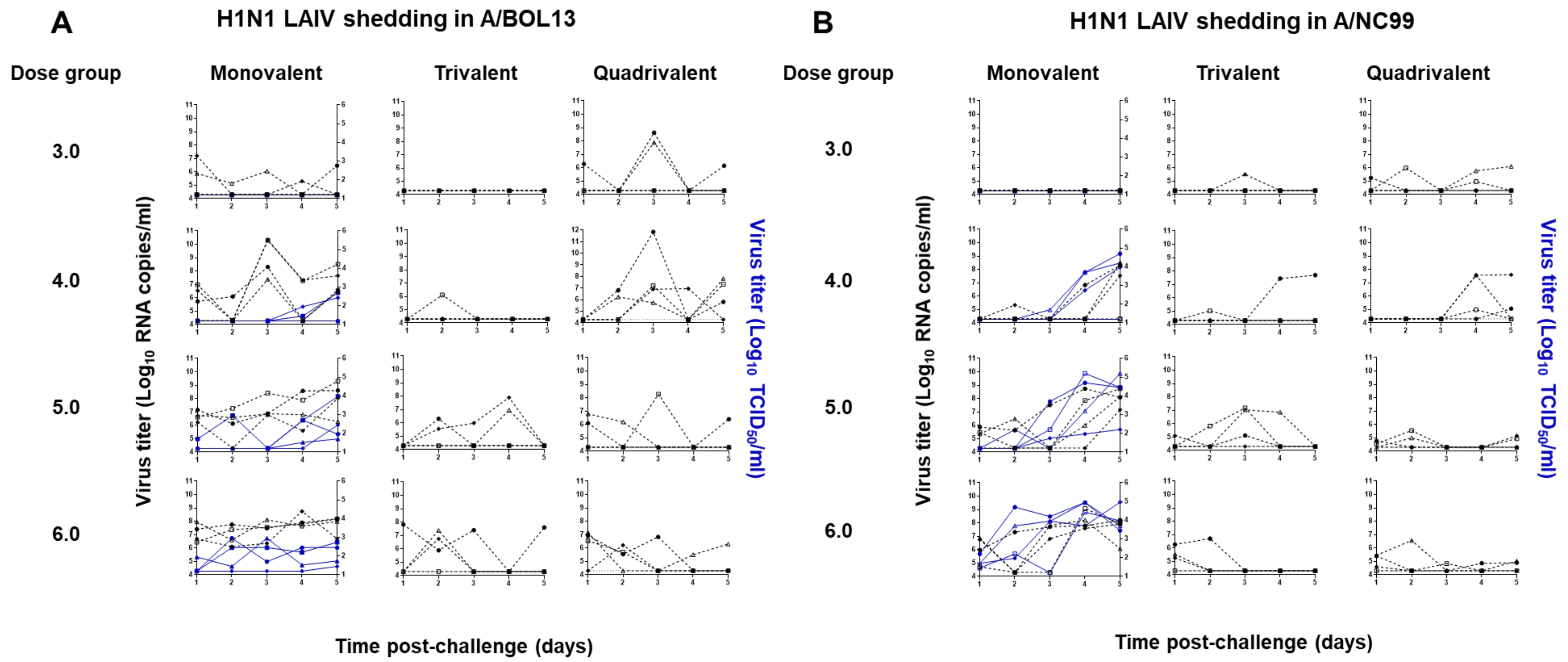

Supplementary Fig. 2. H1N1 LAIV shedding kinetics in individual animals. Days 1–5 post-vaccination, measured by RT-qPCR (left y-axis, dashed black lines) and $TCID_{50}$ (monovalent LAIV animals only, right y-axis, blue lines). Graphs show values from individual animals (symbols). **a** A/BOL13 LAIV shedding in monovalent, trivalent, quadrivalent (right column). **b** A/NC99 LAIV

shedding in monovalent, trivalent, and quadrivalent. Dose groups are aligned vertically, from 3.0–6.0 log_{10} FFU.

A/BOL13, A/Bolivia/559/2013; A/NC99, A/New Caledonia/20/1999; FFU, fluorescent focus units; LAIV, live attenuated influenza vaccine; RT-qPCR, quantitative reverse transcription polymerase chain reaction; $TCID_{50}$, tissue culture infectious dose 50%.

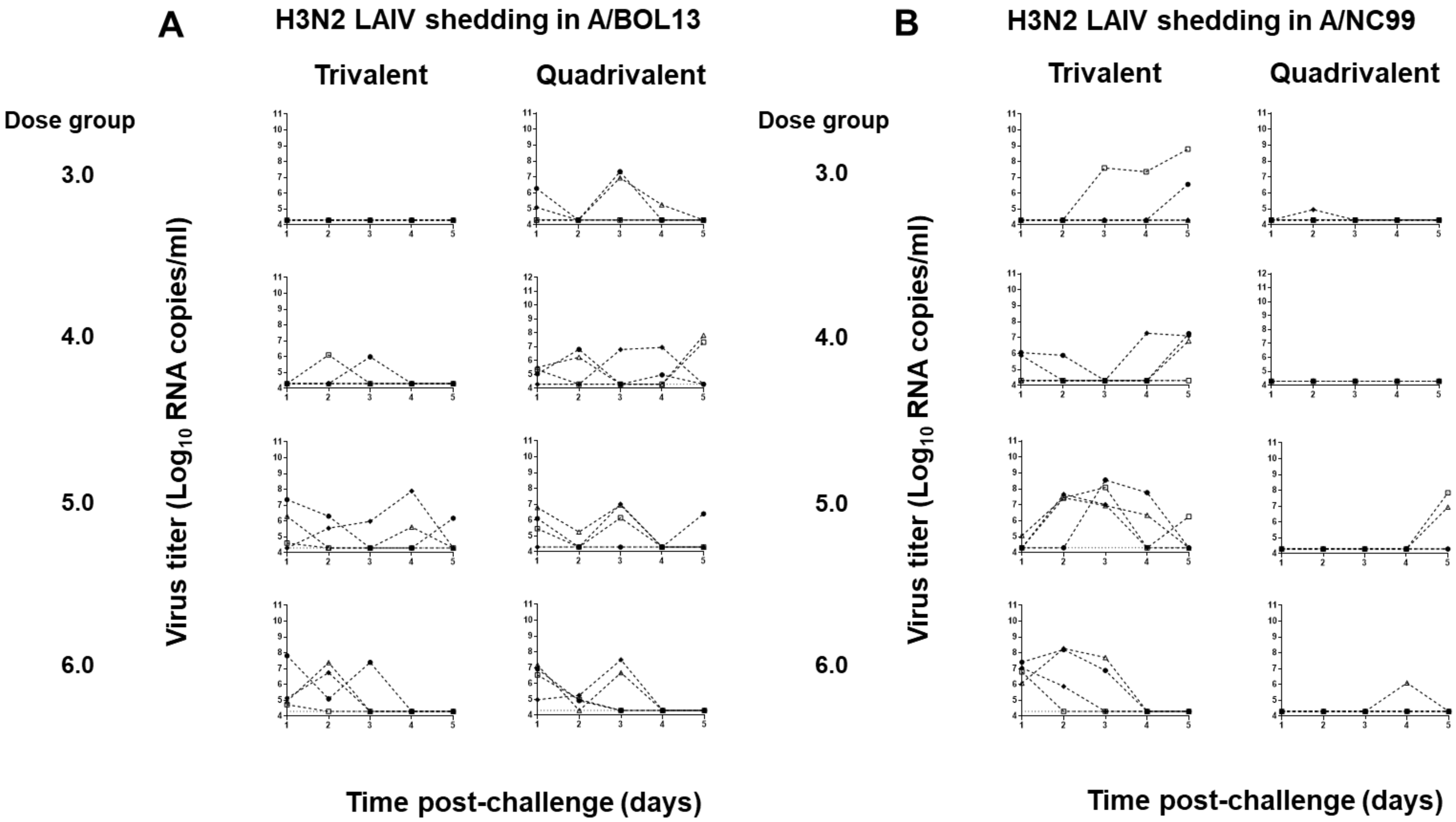

Supplementary Fig. 3. H3N2 LAIV shedding kinetics in individual animals. Days 1–5 post-vaccination, measured by RT-qPCR. Graphs show values from individual animals (symbols). **a–b.** A/SWITZ13 shedding in either A/BOL13 (**a**) or A/NC99 (**b**) trivalent

and quadrivalent LAIV. Dose groups are aligned vertically, from 3.0–6.0 log_{10} FFU.

A/BOL13, A/Bolivia/559/2013; A/NC99, A/New Caledonia/20/1999; FFU, fluorescent focus units; LAIV, live attenuated influenza vaccine; RT-qPCR, quantitative reverse transcription polymerase chain reaction; $TCID_{50}$, tissue culture infectious dose 50%.

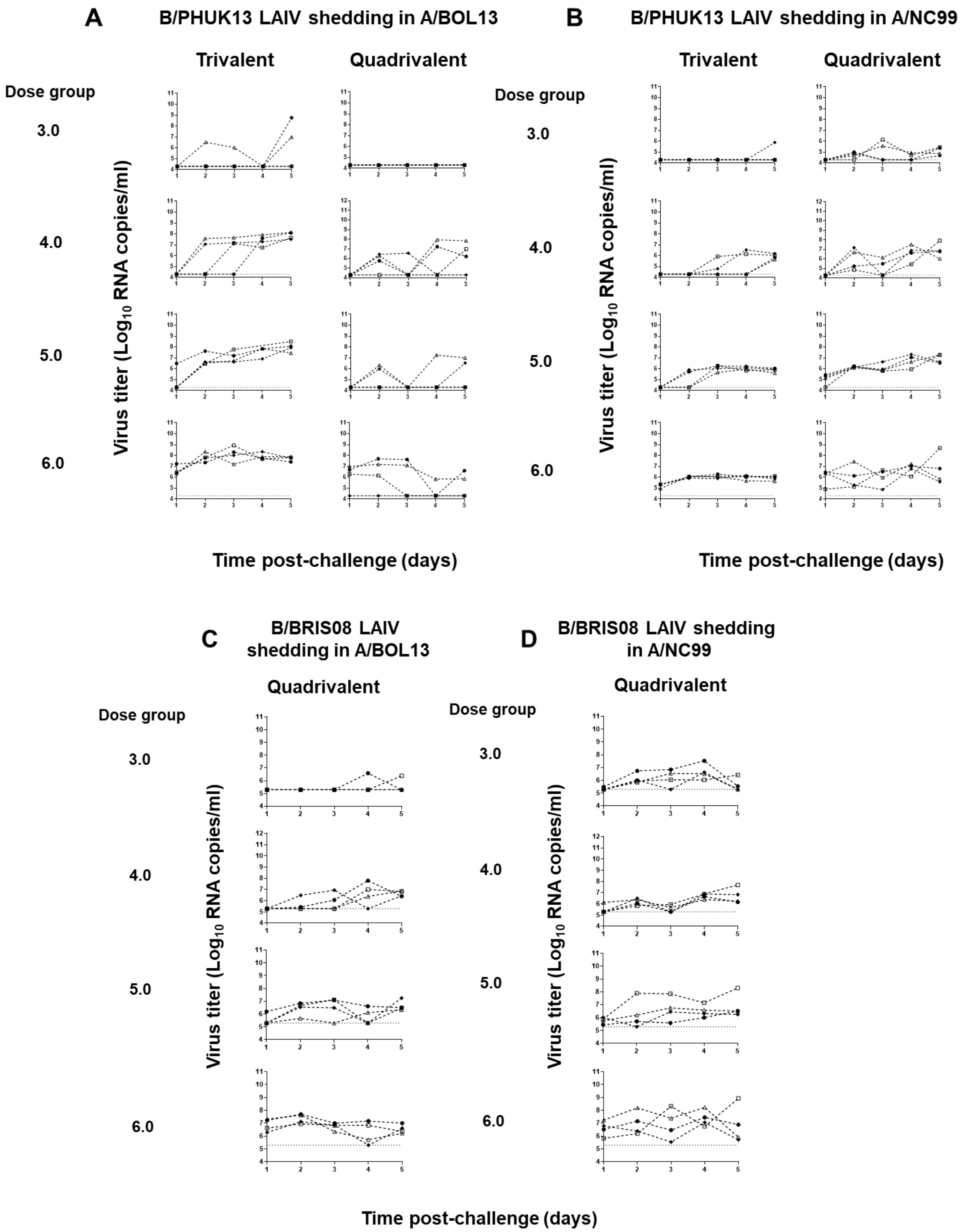

Supplementary Fig. 4. B LAIV shedding kinetics in individual animals. Days 1–5 post-vaccination, measured by RT-qPCR. Graphs show values from individual

animals (symbols). **a–b.** B/PHUK13 LAIV shedding in A/BOL13 (**a**) or A/NC99 (**b**) trivalent and quadrivalent LAIV. **c–d.** B/BRIS08 LAIV shedding in A/BOL13 (**c**) or A/NC99 (**d**) quadrivalent. Dose groups are aligned vertically, from 3.0–6.0 $\log_{10}$ FFU. A/BOL13, A/Bolivia/559/2013; A/NC99, A/New Caledonia/20/1999; B/PHUK13, B/BRIS08, B/Brisbane/60/2008; B/Phuket/3073/2013; FFU, fluorescent focus units; LAIV, live attenuated influenza vaccine; RT-qPCR, quantitative reverse transcription polymerase chain reaction; $TCID_{50}$, tissue culture infectious dose 50%.

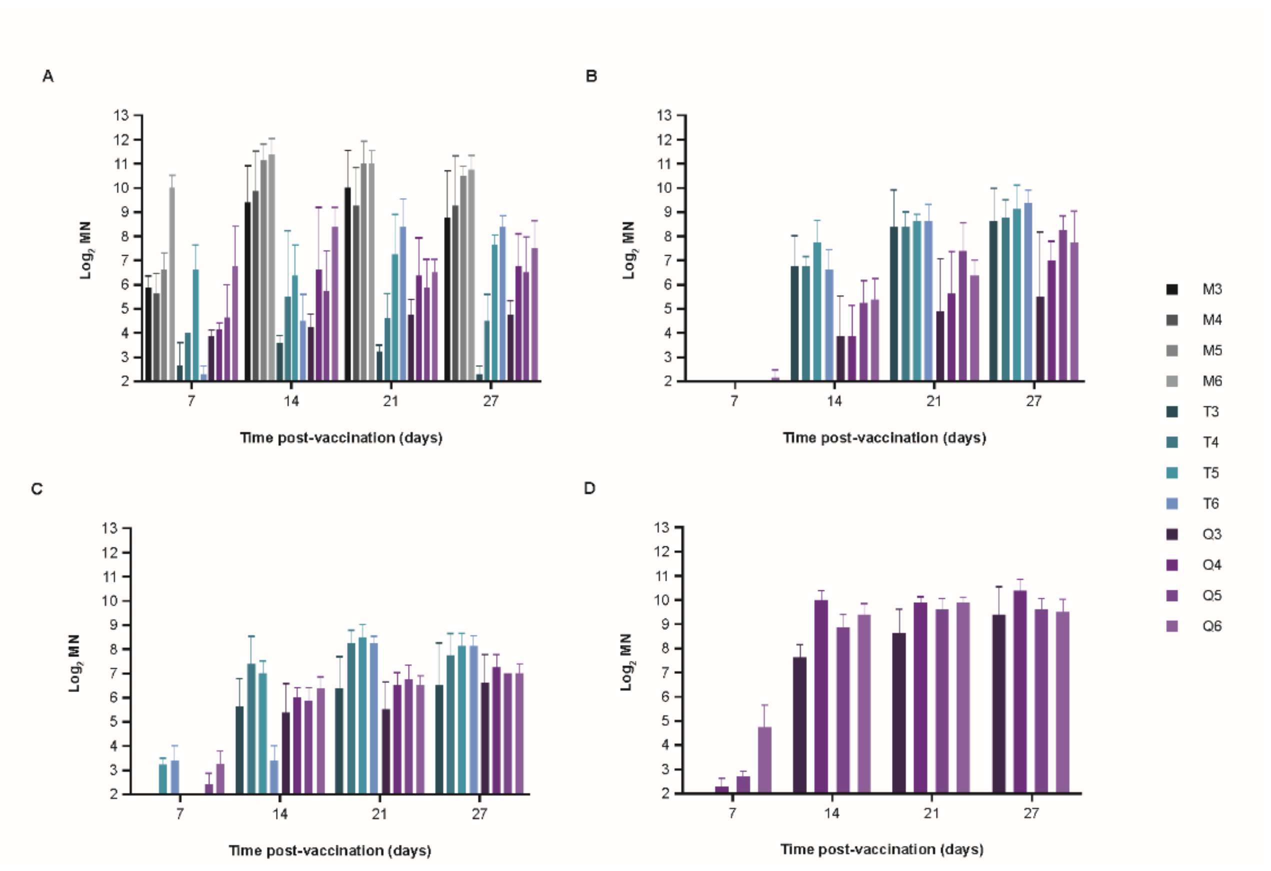
A
B
C
D
Log_2 MN
Time post-vaccination (days)
7
14
21
27
M3
M4
M5
M6
T3
T4
T5
T6
Q3
Q4
Q5
Q6

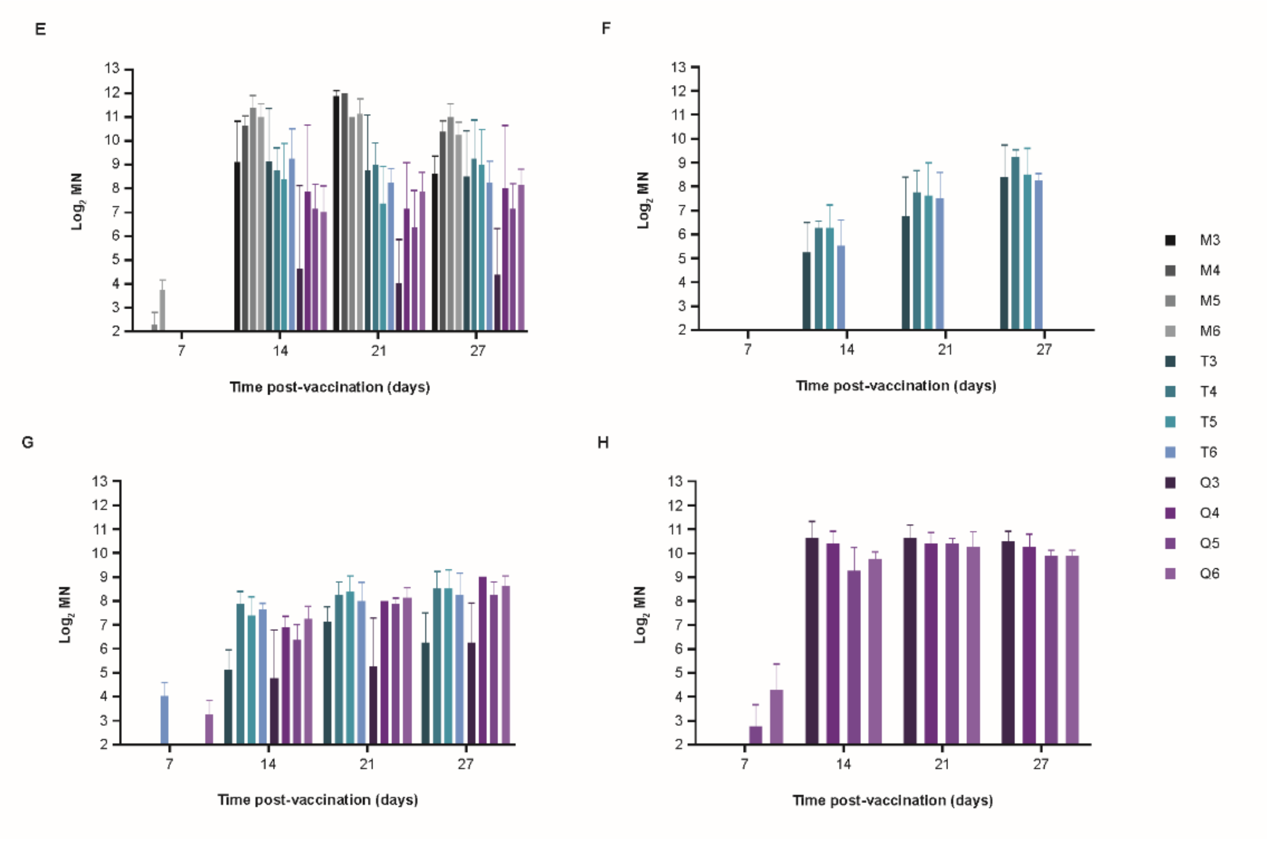
E
F
G
H
Log_2 MN
Time post-vaccination (days)
7
14
21
27
M3
M4
M5
M6
T3
T4
T5
T6
Q3
Q4
Q5
Q6

Supplementary Fig. 5. Serum MN responses to vaccination. MN responses were measured on Days 7, 14, 21, and 27 post-vaccination. *a***–d.** A/BOL13 formulations: **a** A/BOL13; **b** A/SWITZ13; **c** B/PHUK13; **d** B/BRIS08. **e–h** A/NC99 formulations: **e** A/NC99; **f** A/SWITZ13; **g** B/PHUK13; **h** B/BRIS08. Columns and error bars show geometric mean and geometric standard deviation. Grey bars: monovalent LAIV 3.0–6.0 $\log_{10}$ doses (M3–M6), blue bars: trivalent LAIV 3.0–6.0 $\log_{10}$ doses (T3–T6) and purple quadrivalent LAIV 3.0–6.0 $\log_{10}$ doses (Q3–Q6).

A/BOL13, A/Bolivia/559/2013; A/NC99, A/New Caledonia/20/1999; A/SWITZ13, A/Switzerland/9715293/2013; B/BRIS08, B/Brisbane/60/2008; B/PHUK13, B/Phuket/3073/2013; LAIV, live attenuated influenza vaccine; MN, microneutralization.

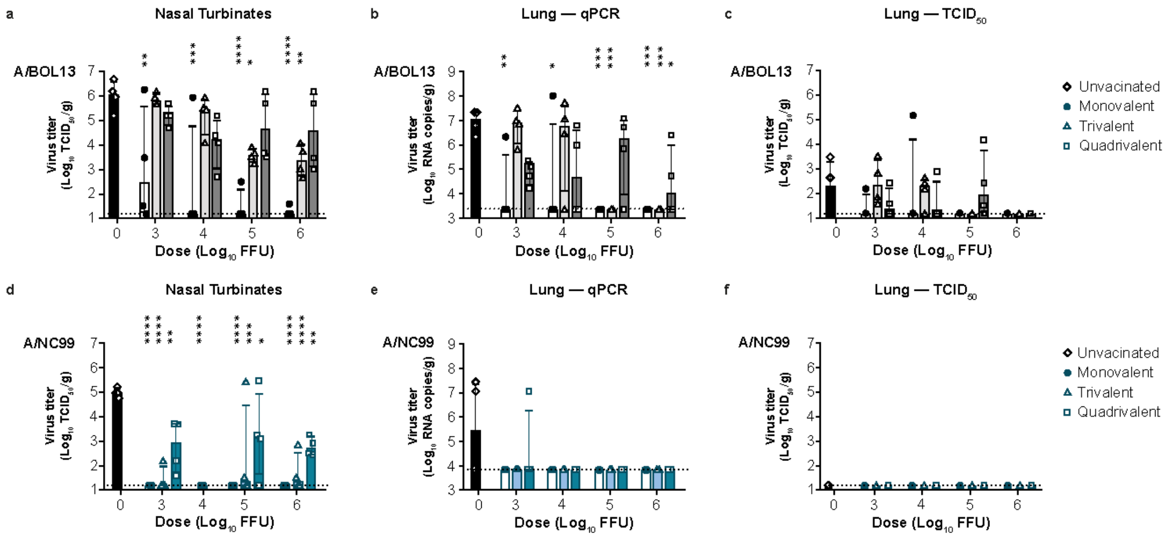

Supplementary Fig. 6. A/BOL13 provides reduced protection against wt challenge virus replication in respiratory tissues. Levels of wt challenge virus replication at Day 3 post-challenge in lung and NT tissues. **a, d**. wt virus titers in NT by $TCID_{50}$. **b, e**. wt virus titers in lungs by RT-qPCR. **c, f**. wt virus titers in lungs by $TCID_{50}$. Data shown are geometric mean virus titers for individual animals (symbols), with 4 animals per dose group. Columns and error bars for all virus titer data show group median and interquartile range. Statistical comparison between groups was performed by two-way analysis of variance with Sidak's post-test correcting for multiple comparisons. **P* < 0.05, ***P* < 0.01, ****P* < 0.001, *****P* < 0.0001. Dotted lines indicate lower limit of detection; values below this were plotted as 0.5x the lower limit of detection.

A/BOL13, A/Bolivia/559/2013; A/NC99, A/New Caledonia/20/1999; FFU, fluorescent focus units; GMT, geometric mean titer; NT, nasal turbinate; RT-qPCR, quantitative reverse transcription polymerase chain reaction; $TCID_{50}$, tissue culture infectious dose 50%; wt, wild-type.

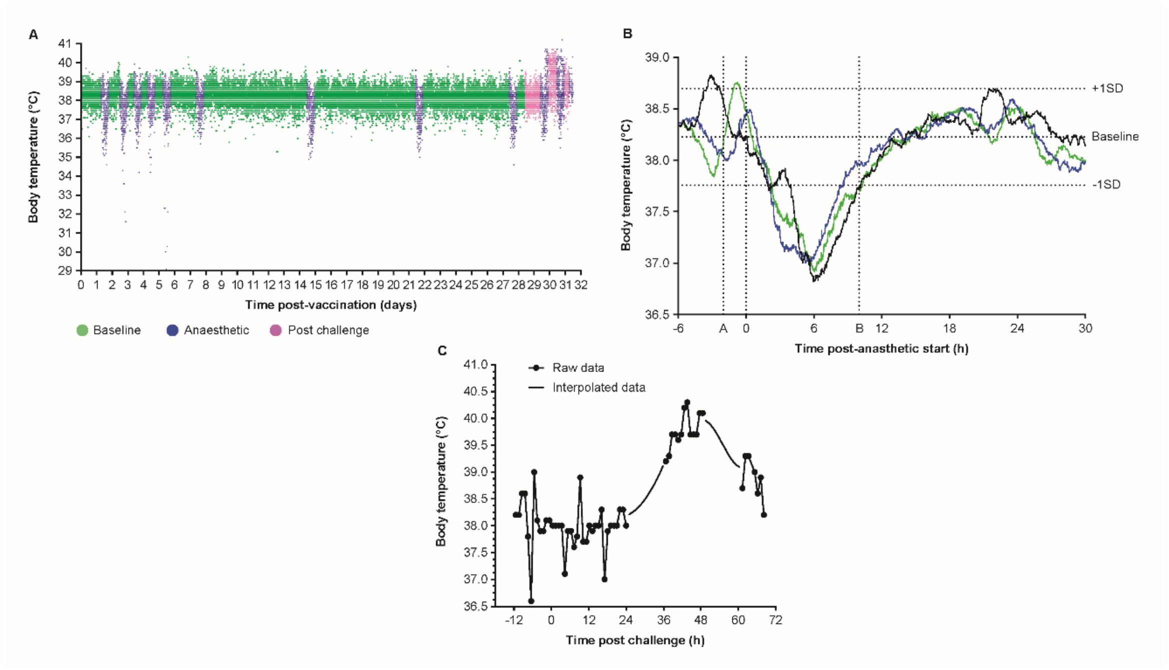

Supplementary Fig. 7. Quantitative analysis of ferret fever development.

Intraperitoneal telemetry monitors were used to measure ferret core temperature 1 hourly for study duration. **a** Example of complete raw temperature data set for wt A/BOL13 challenge (52 animals). Green: normal body temperature. Blue: anesthetic hypothermia. Pink: post-challenge period. **b** Example of removal (A to B) of anesthetic hypothermia artefact, with three representative temperature traces shown (50-point moving average, all animals). **c** Example of quadratic spline interpolation of data to repair excluded hypothermia artefact in post-challenge temperature trace. A/BOL13, A/Bolivia/559/2013; h, hour; SD, standard deviation; wt, wild-type
