# Supplementary material for: Defining the root cause of reduced H1N1 live attenuated influenza vaccine effectiveness: low viral fitness leads to inter-strain competition
Source: NPJ Vaccines. 2021 Mar 12;6:35. doi: 10.1038/s41541-021-00300-z (PMC7955111; doi:10.1038/s41541-021-00300-z)
Supplement: Supplementary file 2 — Reporting Summary [file 41541_2021_300_MOESM2_ESM.pdf]

nature research

Corresponding author(s): Oliver Dibben

Last updated by author(s): Jan 21, 2021

Reporting Summary

Nature Research wishes to improve the reproducibility of the work that we publish. This form provides structure for consistency and transparency in reporting. For further information on Nature Research policies, see our Editorial Policies and the Editorial Policy Checklist.

Statistics

For all statistical analyses, confirm that the following items are present in the figure legend, table legend, main text, or Methods section.

n/a	Confirmed	
☐	☒	The exact sample size (*n*) for each experimental group/condition, given as a discrete number and unit of measurement
☐	☒	A statement on whether measurements were taken from distinct samples or whether the same sample was measured repeatedly
☐	☒	The statistical test(s) used AND whether they are one- or two-sided<br>*Only common tests should be described solely by name; describe more complex techniques in the Methods section.*
☒	☐	A description of all covariates tested
☐	☒	A description of any assumptions or corrections, such as tests of normality and adjustment for multiple comparisons
☐	☒	A full description of the statistical parameters including central tendency (e.g. means) or other basic estimates (e.g. regression coefficient) AND variation (e.g. standard deviation) or associated estimates of uncertainty (e.g. confidence intervals)
☒	☐	For null hypothesis testing, the test statistic (e.g. *F*, *t*, *r*) with confidence intervals, effect sizes, degrees of freedom and *P* value noted<br>*Give P values as exact values whenever suitable.*
☒	☐	For Bayesian analysis, information on the choice of priors and Markov chain Monte Carlo settings
☒	☐	For hierarchical and complex designs, identification of the appropriate level for tests and full reporting of outcomes
☒	☐	Estimates of effect sizes (e.g. Cohen's *d*, Pearson's *r*), indicating how they were calculated

Our web collection on statistics for biologists contains articles on many of the points above.

Software and code

Policy information about availability of computer code

Data collection	Not applicable
Data analysis	Not applicable

For manuscripts utilizing custom algorithms or software that are central to the research but not yet described in published literature, software must be made available to editors and reviewers. We strongly encourage code deposition in a community repository (e.g. GitHub). See the Nature Research guidelines for submitting code & software for further information.

Data

Policy information about availability of data

All manuscripts must include a data availability statement. This statement should provide the following information, where applicable:

- Accession codes, unique identifiers, or web links for publicly available datasets
- A list of figures that have associated raw data
- A description of any restrictions on data availability

All data associated with this study are available in the main text or the supplementary materials. Further information and requests for resources and reagents should be directed to and will be fulfilled by the Lead Contact, Oliver Dibben (oliver.dibben@astrazeneca.com).

Field-specific reporting

Please select the one below that is the best fit for your research. If you are not sure, read the appropriate sections before making your selection.

☒ Life sciences ☐ Behavioural & social sciences ☐ Ecological, evolutionary & environmental sciences

For a reference copy of the document with all sections, see nature.com/documents/nr-reporting-summary-flat.pdf

Life sciences study design

All studies must disclose on these points even when the disclosure is negative.

Sample size	No formal sample size calculation was performed. Groups of 4 animals per group was based on prior observations where at least 3 animals per group were necessary to generate statistically robust observations. A small number of temperature data loggers had been seen to become corrupted, requiring 4 animals per group to allow for this.
Data exclusions	No data were excluded from any analyses unless data could not be generated. Temperature data files from two animals were corrupted and so could not be used - stated in the manuscript. Due to insufficient sample volume, two groups of 4 animals did not have TCID50 data generated from nasal turbinate tissues, although this was not a primary endpoint - also stated in manuscript.
Replication	In subsequent, independent studies continuing our investigation of LAIV efficacy in the ferret model - not described in this manuscript - the key finding shown here has been reproduced: that using a low vaccine dose, the fitter A/NC99 H1N1 strain provides protection from challenge in QLAIV formulation where the less fit A/BOL13 does not.
Randomization	Ferrets of mixed age and sex were randomly distributed into study groups using a randomizing matrix in Microsoft Excel.
Blinding	As this was not a clinical study and data were required to be generated and shared incrementally as the work progressed, blinding would not have been feasible and was not used.

Reporting for specific materials, systems and methods

We require information from authors about some types of materials, experimental systems and methods used in many studies. Here, indicate whether each material, system or method listed is relevant to your study. If you are not sure if a list item applies to your research, read the appropriate section before selecting a response.

Materials & experimental systems

n/a	Involved in the study	
☐	☒	Antibodies
☐	☒	Eukaryotic cell lines
☒	☐	Palaeontology and archaeology
☐	☒	Animals and other organisms
☒	☐	Human research participants
☒	☐	Clinical data
☒	☐	Dual use research of concern

Methods

n/a	Involved in the study	
☒	☐	ChIP-seq
☒	☐	Flow cytometry
☒	☐	MRI-based neuroimaging

Antibodies

Antibodies used	Anti-HA protein primary antibodies, either obtained from the National Institute for Biological Standards and Control, UK (NIBSC), or produced internally at AstraZeneca.
Validation	Suitability of primary antibodies for quantification of LAIV strains was conducted internally, confirming cross-reactivity with the specific strain of interest and a lack of cross-reactivity with other virus subtypes in multivalent formulation.

Eukaryotic cell lines

Policy information about cell lines

Cell line source(s)	Madin–Darby canine kidney (MDCK) cells, used in FFA, TCID50 and MN assays, were obtained from ATCC and passaged fewer than 20 times prior to use in analytical tests.
Authentication	No other cell line authentication was conducted.
Mycoplasma contamination	MDCK cells were confirmed mycoplasma negative at the time of cell bank generation. Cells were not mycoplasma tested again post-thaw, but were subject to a limited number of passages as described in the materials and methods.

Commonly misidentified lines (See ICLAC register)	N/A

Animals and other organisms

Policy information about studies involving animals; ARRIVE guidelines recommended for reporting animal research

Laboratory animals	Studies were conducted in outbred, mixed sex, specific pathogen free, 14–26-week-old ferrets (Mustela putorius furo; Charles River Laboratories Ltd, Ballina, Ireland).
Wild animals	The study did not involve wild animals.
Field-collected samples	The study did not involve samples collected from the field.
Ethics oversight	Ethical approval of the study was provided by AstraZeneca's Council for Science & Animal Welfare (C-SAW) prior to study start.

Note that full information on the approval of the study protocol must also be provided in the manuscript.
